# Mannose Promotes β‐Amyloid Pathology by Regulating BACE1 Glycosylation in Alzheimer's Disease

**DOI:** 10.1002/advs.202409105

**Published:** 2025-01-14

**Authors:** Chensi Liang, Ziqi Yuan, Shangchen Yang, Yufei Zhu, Zhenlei Chen, Dan Can, Aiyu Lei, Huifang Li, Lige Leng, Jie Zhang

**Affiliations:** ^1^ Fujian Provincial Key Laboratory of Neurodegenerative Disease and Aging Research Institute of Neuroscience School of Medicine Xiamen University Xiangan South Road Xiamen Fujian 361102 P. R. China; ^2^ Department of Pediatrics Xiamen Maternity and Child Health Hospital affiliated to Xiamen University Zhenhai Road Xiamen Fujian 361003 P. R. China; ^3^ The Key Laboratory of Neural and Vascular Biology Ministry of Education Hebei Medical University Zhongshan East Road Shijiazhuang Hebei 050017 P. R. China; ^4^ Institute of Neuroscience Fujian Medical University Xueyuan Road Fuzhou Fujian 350122 P. R. China

**Keywords:** alzheimer's disease, BACE1, glycosylation, mannose, β‐amyloid

## Abstract

Hyperglycemia accelerates Alzheimer's disease (AD) progression, yet the role of monosaccharides remains unclear. Here, it is demonstrated that mannose, a hexose, closely correlates with the pathological characteristics of AD, as confirmed by measuring mannose levels in the brains and serum of AD mice, as well as in the serum of AD patients. AD mice are given mannose by intra‐cerebroventricular injection (ICV) or in drinking water to investigate the effects of mannose on cognition and AD pathological progression. Chronic mannose overload increases β‐amyloid (Aβ) burdens and exacerbates cognitive impairments, which are reversed by a mannose‐free diet or mannose transporter antagonists. Mechanistically, single‐cell RNA sequencing and metabolomics suggested that mannose‐mediated N‐glycosylation of BACE1 and Nicastrin enhances their protein stability, promoting Aβ production. Additionally, reduced mannose intake decreased BACE1 and Nicastrin stability, ultimately lowering Aβ production and mitigating AD pathology. this results highlight that high‐dose mannose consumption may exacerbate AD pathogenesis. Restricting dietary mannose may have therapeutic benefits.

## Graphical abstract

Mannose levels elevate in the brains of Alzheimer's disease (AD) patients and AD mice. Chronic overload of mannose increases Aβ burdens and exacerbates cognitive impairments, which can be reversed through a mannose‐free diet or the use of mannose transporter antagonists. Mechanistically, mannose‐mediated N‐glycosylation of BACE1 and Nicastrin enhances their protein stability, further promoting Aβ production. Restricting dietary mannose may offer therapeutic benefits for AD patients.

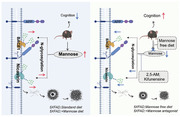

## 1 Introduction

Alzheimer's disease (AD) is characterized by progressive cognitive decline and brain atrophy associated with the accumulation of β‐amyloid (Aβ) plaques and tau protein neurofibrillary tangles (NFTs). Targeting the pathogenesis of Aβ is an effective intervention for AD. The regulatory mechanism underlying Aβ generation deserves detailed investigation. Brain energy dysregulation is closely involved in the pathogenesis of AD.^[^ ^1^, ^2^ ^]^ The progressive decline in carbohydrate metabolism efficiency weakens neurons, making them more susceptible to toxic damage.^[^ ^3^ ^]^

One important process that becomes dysfunctional in AD is carbohydrate metabolism.^[^ ^4^ ^]^ Most monosaccharides can cross the blood‐brain barrier and affect brain glycolysis, especially five major monosaccharides: D‐glucose, D‐mannose, D‐fructose, L‐fucose, and L‐galactose. Glucose enters the brain from the vasculature through highly efficient glucose transporters and requires insulin for optimal cellular utilization.^[^ ^5^ ^]^ The effects of D‐glucose on the pathogenesis of AD have been well studied. Impairment of cerebral glucose metabolism is considered a hallmark of AD, often preceding cognitive dysfunction by decades in patients.^[^ ^1^, ^6^, ^7^ ^]^ However, little is known about the effects of other monosaccharides in AD. Therefore, elucidating the specific role of carbohydrate metabolism, including insulin signaling pathways, in both the pathogenesis and interventions for AD holds significant value for future translational studies and clinical applications.

Monosaccharides not only influence metabolism but also impact protein glycosylation in AD. Glycosylation is a common form of posttranslational protein modification including N‐linked and O‐linked glycans. Glycosylation is achieved through the assembly of a total of 41 bonds between 13 different monosaccharides—glucose, mannose, galactose, fucose, etc. Among them, mannose‐rich N‐glycosylation has been reported to have multiple roles during the pathogenesis of AD, including extracellular matrix dysfunction, neuroinflammation, synaptic dysfunction, cell adhesion alteration, lysosomal dysfunction, endocytic trafficking dysregulation, endoplasmic reticulum dysfunction.^[^ ^8^ ^]^ The sugar chain of N‐glycans contains a common pentasaccharide core composed of mannose and N‐acetylglucosamine. Despite the crucial role of mannose in protein glycosylation, research on mannose and AD remains limited.

Mannose is widely distributed in body fluids and tissues, especially in the nerve system, retina, and intestines. It is used to synthesize glycoproteins and participate in immune regulation. Gonzalez et al.^[^ ^9^ ^]^ found that mannose inhibits tumor cell growth and decreases glucose levels by inhibiting hexokinases and phosphoglucose isomerase. Additionally, mannose has been shown to alleviate intestinal inflammation,^[^ ^10^ ^]^ and serve as a drug adjuvant.^[^ ^11^ ^]^ Despite its beneficial effects in the peripheral nervous system, mannose appears to play a detrimental role in the central nervous system. Xu et al.^[^ ^12^ ^]^ reported that a diet rich in mannose could induce cognitive impairment and anxiety‐like behaviors in mice. In addition, high plasma D‐mannose levels are closely associated with insulin resistance, which is a risk factor for AD.^[^ ^13^, ^14^ ^]^ D‐Mannose may also damage microglial function, potentially accelerating AD pathogenesis.^[^ ^15^ ^]^ However, the precise mechanisms underlying the effects of mannose on AD remain unclear.

Protein N‐glycosylation is vital for the pathogenesis of AD. Multiple AD pathogenic proteins such as Amyloid Precursor Protein (APP),^[^ ^16^ ^]^ BACE1, and Nicastrin,^[^ ^17^, ^18^ ^]^ can all be N‐glycosylated. N‐glycosylation is required for the function of these proteins. Mannose is the C2 epimer of glucose and a component of glycoconjugates.^[^ ^19^ ^]^ Mannose can freely enter cells, but the transporter responsible for its uptake is unknown. Mannose may possibly share the same sugar transporter as glucose.^[^ ^20^ ^]^ Mannose can be phosphorylated to mannose‐6‐phosphate (M‐6‐P) by hexokinase. M‐6‐P can then undergo further isomerization to fructose‐6‐phosphate (F‐6‐P) by phosphomannose isomerase (PMI). F‐6‐P can be further converted to fructose‐1,6‐bisphosphate (FBP) by phosphofructokinase‐1 (PFK‐1). Additionally, M‐6‐P can be converted to mannose‐1‐phosphate (M‐1‐P) by phosphomannomutase (PMM). GDP‐mannose pyrophosphorylase (GPP) catalyzes the reaction between GTP and M‐1‐P to produce GDP‐mannose.^[^ ^21^ ^]^

In this study, we found that the serum and brain mannose levels increased in AD patients and AD mice. Mannose supplementation exacerbates the Aβ burden and cognitive impairments in AD mice. We also designed the mannose‐free diet and found that AD mice fed with this diet exhibited lower Aβ burdens. The mannose transporter antagonist (2,5‐anhydro‐D‐mannose (2,5‐AM)), and the α‐mannosidase inhibitor kifunensine (Kif) can both ameliorate cognitive impairments in AD mice. Mechanistically, mannose promotes the stability of the β‐ and γ‐secretase subunits, BACE1 and Nicastrin, through high‐mannose N‐glycosylation.

## 2 Results

### 2.1 Mannose Increases in AD Mice and Patients

**Figure 1:**
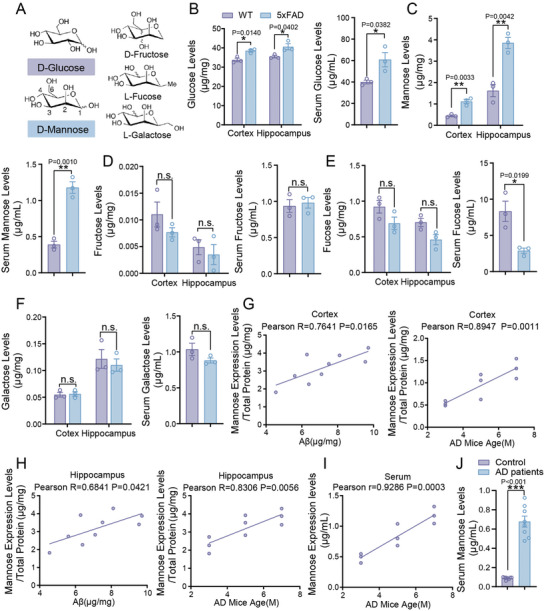
Mannose levels are increased in response to pathology feature of AD. A) Structural of D‐Glucose, D‐mannose D‐fructose, L‐fucose, and L‐galactose. B) The glucose levels in cortex, hippocampus and serum of 7‐month‐old wildtype and 5×FAD mice. *n* = 3 mice. C) The mannose levels in cortex, hippocampus and serum of 7‐month‐old wildtype and 5×FAD mice. *n* = 3 mice. D) The fructose levels in cortex, hippocampus and serum of 7‐month‐old wildtype and 5×FAD mice. *n* = 3 mice. E) The fucose levels in cortex, hippocampus and serum of 7‐month‐old wildtype and 5×FAD mice. *n* = 3 mice. F) The galactose levels in cortex, hippocampus and serum of 7‐month‐old wildtype and 5×FAD mice. *n* = 3 mice. G) Correlation analysis of mannose levels in cortex of 3/5/7‐month‐old 5×FAD mice with age and Aβ1‐42 levels. *n* = 3 mice. H) Correlation analysis of mannose levels in hippocampus of 3/5/7‐month‐old 5×FAD mice with age and Aβ1‐42 levels. *n* = 3 mice. I) Correlation analysis of mannose levels in serum of 3/5/7‐month‐old 5×FAD mice with age and Aβ1‐42 levels. *n* = 3 mice. J) Mannose levels in serum from AD patients and controls were determined by ELISA. AD patients: *n* = 8; Control: *n* = 8. Data represent mean ± SEM, n.s.: not significant, ^*^ *p* < 0.05, ^**^ *p* < 0.01, ^***^ *p* < 0.001, unpaired t test for behavioral statistics. Other statistical applications were analyzed by one‐way ANOVA with Tukey's post hoc analysis.

The dysregulation of glycometabolism is a feature of the pathogenesis of AD. The major monosaccharides found in commonly consumed carbohydrates are D‐glucose, D‐mannose, D‐fructose, L‐fucose, and L‐galactose (**Figure** **1**A). We first measured the levels of these five monosaccharides in the brain and serum of 7‐month‐old 5×FAD mice and littermate controls. Notably, glucose and mannose both increased in the hippocampus/cortex and serum of AD mice compared with the control (Figure 1B,C). The other three monosaccharides (D‐fructose, L‐fucose, and L‐galactose) did not change in AD samples compared with control, except for the serum fucose level, which decreased in AD mice (Figure 1D–F). To investigate the correlations between different monosaccharides, we reviewed available databases and identified that microglia, given their close link with metabolic processes, provide the most extensive datasets related to glucose, mannose, and fructose metabolism. Consequently, we analyzed metabolic genes related to these sugars in diabetic and microglial databases to offer initial insights. We selected microglia for analysis due to their central role in neuroinflammation and metabolic regulation in the brain, as well as their relevance in Alzheimer's disease (AD) pathology. Microglia are not only highly metabolically active but also exhibit significant responsiveness to changes in glucose and other monosaccharide levels, influencing both amyloid processing and neuroinflammatory pathways.^[^ ^22^ ^]^ Furthermore, extensive datasets and established models for microglial glucose metabolism enabled a more detailed exploration of monosaccharide‐related metabolic dynamics, compared with other brain cell types for which comparable data are limited.^[^ ^23^, ^24^ ^]^ We conducted an analysis of RNA‐seq and ChIP‐seq of microglia, as well as ChIP‐seq data of diabetes. We found a strong positive correlation between mannose and glucose levels in AD‐ related changes in glycometabolism, with consistent trends in their changes, but their metabolism does not converge with fructose (Figure S1A–C, Supporting Information). The up‐regulation of mannose in AD draws our attention. To further investigate the correlation of mannose level with the pathogenesis of AD, we measured the mannose and Aβ levels in the cortex/hippocampus and serum of 3‐, 5‐, and 7‐month‐old AD mice using Enzyme‐Linked Immunosorbent Assay (ELISA). We found that the mannose levels are positively correlated with both age and Aβ levels (Figure 1G–I). Clinically, the levels of mannose are much higher in the serum of AD patients compared with age‐matched healthy individuals (Figure 1J).

### 2.2 Mannose Exacerbates the Aβ Burden and Cognitizve Impairment of AD Mice, Causes Cognitive Impairment in Wild Type Mice

**Figure 2:**
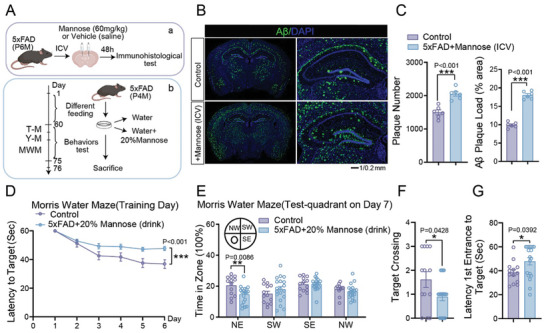
Long‐term intake of mannose could aggravate the pathological process of 5×FAD and cognitive impairment. A) Schematic diagram of ICV administration of mannose or vehicle in 5×FAD mice is shown in (a), 48 h after injection, the mice brain slice were subjected to immunostaining. Schematic diagram of administration of 20% mannose or vehicle in 5×FAD mice is shown in (b). B, C) Immunofluorescence staining of Aβ in cerebrum from 6‐month‐old 5×FAD mice treated with mannose or control vehicle (saline) by ICV. Representative confocal images are shown on panel (B), Scale bar:1 mm, 0 .2 mm. Quantitation of plaque number and load of cortex and hippocampus are showed in (C), *n* = 6 mice. D–G) Morris water maze tests of 6‐month‐old 5×FAD mice drinking water with or without 20% mannose were analyzed for escape latency during a 6‐day training period (D), the time spent in the target zone and other quadrants (southwest, southeast, and northwest) (E), number of target crossings (F) and time required from entrance to the target platform (G) were measured on the next day. Mouse number used in behavior tests: 5×FAD mice: *n* = 13 mice, 5×FAD mice drinking 20% mannose: *n* = 19 mice.Data represent mean ± SEM, n.s.: not significant, ^*^ *p* < 0.05, ^**^ *p* < 0.01, ^***^ *p* < 0.001, unpaired t test for behavioral statistics. Other statistical applications were analyzed by one‐way ANOVA with Tukey's post hoc analysis.

Considering the enhanced mannose levels in AD, we wondered whether extra mannose administration would affect the pathogenesis of AD. We first injected mannose (60 mg kg^−1^) into the lateral ventricle of 6‐month‐old 5×FAD mice (**Figure** **2**A‐a). The brain slices were collected for Aβ staining 48 h later. We found that mannose injection significantly increased the amyloid plaque burden in 5×FAD mice (Figure 2B,C). To determine whether the cognitive effects of mannose on AD mice are influenced by sex, we conducted four groups of 6‐month‐old 5×FAD mice administering stereotactic injections of mannose (60 mg kg^−1^) or vehicle (saline): saline‐treated males, saline‐treated females, mannose‐treated males, and mannose‐treated females. Brain sections were collected 48 h after treatment for immunofluorescence analysis (Figure S2A, Supporting Information). Several studies have reported sex differences in Aβ deposition in 5×FAD mice, with females generally exhibiting a higher amyloid burden than males.^[^ ^25^, ^26^ ^]^ Interestingly, our further literature review revealed that these differences are brain‐region specific. For example, research by Lansdell et al.^[^ ^27^ ^]^ found no significant difference in cortical Aβ burden between male and female 5×FAD mice at 6 months of age (% immunoreactive area; male: 4.057 ± 0.4897 vs female: 6.350 ± 1.961, ANOVA, *p* = 0.1289). Similarly, Poon et al.^[^ ^28^ ^]^ reported that while female 5×FAD mice exhibit higher Aβ levels in the dentate gyrus (DG) compared to males (^*^ *p* < 0.05), no significant differences were observed in other hippocampal regions such as the subiculum (SUB), CA1‐2, and CA3. Based on these findings, we quantified Aβ plaque burden across different regions of the hippocampus, including the CA1‐3, and dentate gyrus (DG), in the four groups. Our results showed the following: in the vehicle control groups, no significant differences in Aβ burden were observed between male and female 5×FAD mice in the CA1, CA2, or DG. However, female mice exhibited higher Aβ levels in the CA3 compared to males. Following mannose administration, Aβ plaque burden increased in CA1, CA3, and DG in both male and female mice, but on difference in CA2. Comparing the mannose‐treated groups, no significant sex differences were observed in the CA1, CA2, or DG. However, in the CA3, female mice continued to exhibit a higher Aβ burden than male mice (Figure S2B–D, Supporting Information).

To closely mimic the mannose uptake, 4‐month‐old 5×FAD mice were given 20% mannose in drinking water for 2 months (Figure 2A‐b). Mannose levels in the brain and serum were significantly increased by oral mannose administration (Figure S2E,F, Supporting Information), without affecting water intake volume (Figure S2G, Supporting Information) or body weight (Figure S2H,I, Supporting Information). The mice were subjected to the water maze for cognitive behavioral test. Notably, extra mannose administration exacerbates the cognitive impairment of 5×FAD mice (Figure 2D–G). These data suggested that overload of mannose by direct brain injection or oral administration both aggravated the pathogenesis of AD. To explore whether a 20% mannose diet also affects cognitive function in wildtype mice, we first gave 20% mannose in drinking water to the wild‐type mice for 2 months and then conducted cognitive related behavioral tests (Figure S3A, Supporting Information). After 2 months drinking of 20% mannose, the level of mannose in the serum and brain of mice did increase, but there was no significant difference in body weight (Figure S3B, C, Supporting Information). The results of behavioral tests showed that drinking 20% mannose affected the spatial memory ability of mice (Figure S3D–I, Supporting Information). These results suggest that high‐dose mannose intake may indeed impair cognitive function in wild‐type mice over time.

### 2.3 Mannose‐Free Diet Alleviated the Cognitive Impairment of 5×FAD Mice

**Figure 3:**
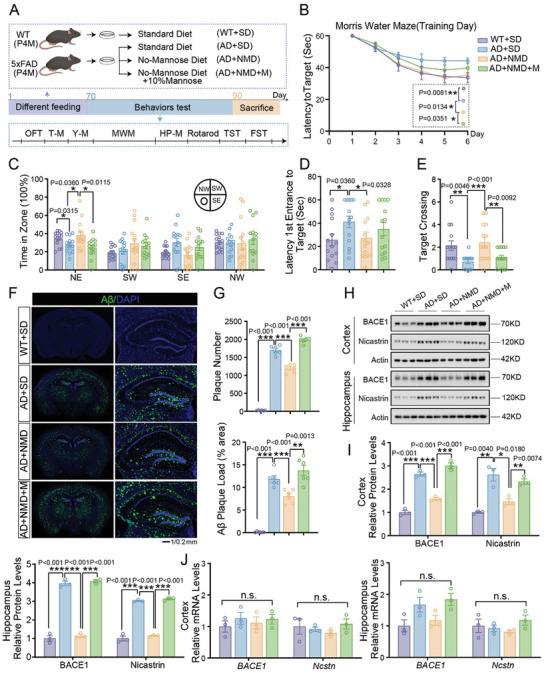
Mannose‐free diet alleviated the cognitive impairment of 5×FAD mice. A) Schematic diagram of different diet for wildtype mice and 5×FAD mice (for 70 days). B–E) Morris water maze tests of 6‐month‐old wildtype mice with standard diet, 5×FAD mice with standard diet, 5×FAD mice with no‐mannose diet and 5×FAD mice with no‐mannose diet+10% mannose mice were analyzed for escape latency during a 6‐day training period (B), the time spent in the target zone and other quadrants (southwest, southeast, and northwest) (C), time required from entrance to the target platform (D) and number of target crossings (E) were measured on the next day. F, G) Immunofluorescence staining of Aβ from 6‐month‐old 5×FAD mice with standard diet, 5×FAD mice with no‐mannose diet and 5×FAD mice with no‐mannose diet+10% mannose. Representative confocal images are shown on panel (F), Scale bar:1 mm, 0 .2 mm. Quantitation of plaque number and diameter are showed in (G), *n* = 6 mice. H, I) Western blot analysis of BACE1 and Nicastrin expression in cortex and hippocampus of 6‐month‐old wildtype mice with standard diet, 5×FAD mice with standard diet, 5×FAD mice with no‐mannose diet and 5×FAD mice with no‐mannose diet+10% mannose mice. Quantification of relative protein levels are shown in (I), *n* = 3 mice. L) *BACE1* and *Ncstn* expression were measured in cortex and hippocampus of 6‐month‐old wildtype mice with standard diet, 5×FAD mice with standard diet, 5×FAD mice with mannose‐free diet and 5×FAD mice with mannose‐free diet+10% mannose mice by real‐time PCR, *n* = 3 mice. Mouse number used in behavior tests: Wild type mice with standard diet: *n* = 14 mice, 5×FAD mice with standard diet: *n* = 14 mice, 5×FAD mice with no‐mannose diet: *n* = 15 mice, 5×FAD mice with no‐mannose diet+10% mannose: *n* = 15 mice. Data represent mean ± SEM, n.s.: not significant, ^*^ *p* < 0.05, ^**^ *p* < 0.01, ^***^ *p* < 0.001, unpaired t test for behavioral statistics. Other statistical applications were analyzed by one‐way ANOVA with Tukey's post hoc analysis.

The regular mouse chow contains mannose that may participate in the pathogenesis of AD. To further investigate the effect of mannose in regular mouse chow, we customized mannose‐free mouse chow. The mannose free chow contains 0.6 µg g^−1^ mannose, which is much lower than the mannose level in standard mouse chow (4.3 µg g^−1^) (Figure S4A, Supporting Information). Other metabolizable energy sources are comparable (Figure S4B, Supporting Information). 4‐month‐old 5×FAD mice or WT mice were given the standard chow, mannose‐free chow, or mannose‐free chow with 10% mannose in drinking water for 70 days before the following tests (**Figure** **3**A). We first confirmed that the mannose levels in the hippocampus/cortex and serum were decreased in 5×FAD mice fed with mannose‐free chow compared with standard chow, and the extra mannose in drinking water increased the brain and serum mannose levels (Figure S4C, Supporting Information) without affecting the body weight (Figure S4D, Supporting Information).

These mice were then subjected to behavioral tests. We found that the mannose‐ free diet attenuated the learning and cognitive impairment of 5×FAD mice measured by water maze tests (Figure 3B–E), the motor activity, anxiety‐ and depression related behaviors were not affected by mannose‐free chow feed tested by an open field, rotarod, high plus maze, tail suspension and forced swimming tests (Figure S4E–J, Supporting Information). 5×FAD mice fed with mannose‐free chow showed a remarkable reduction of Aβ deposition in the brain, however, an extra 10% mannose in drinking water reversed this reduction (Figure 3F,G). The lowered Aβ deposition suggested the APP processing pathway may be affected by mannose free chow. We found that the protein levels of BACE1 and Nicastrin in the hippocampus and cortex were significantly decreased in 5×FAD mice fed mannose‐free chow compared with standard chow feeding (Figure 3H,I). The mRNA levels of BACE1 and Nicastrin were not affected (Figure 3J).

### 2.4 The Mannose Analog 2,5‐AM Attenuates the Pathology and Cognitive Impairments in AD Mice

**Figure 4:**
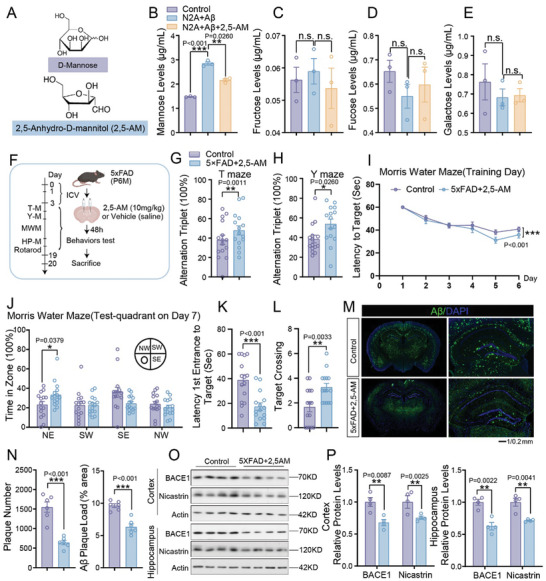
2,5‐AM relieved the cognitive impairment of 5×FAD mice. A) Structural of D‐Mannose and 2,5‐AM. B–E) Mannose (B) /fructose (C) /fucose (D) /galactose (E) levels of N2A cells treated with mannose with or without 2,5‐AM were detected by ELISA kit. *n* = 3 experimental replication/group. F) Schematic diagram of giving 2,5‐AM (10 mg kg^−1^) or control vehicle (saline) ICV on 6‐month‐old 5×FAD mice. G–L) Behavioral analysis of 6‐month‐old 5×FAD mice and 5×FAD+2,5‐AM mice by T maze (G), Y maze (H) and Morris water maze tests (I–L). M,N) Immunofluorescence staining of Aβ in cerebrum from 6‐month‐old 5×FAD mice and 5×FAD+2,5‐AM mice. Representative confocal images are shown on panel (M), Scale bar:1 mm, 0 .2 mm. Quantitation of plaque number and load are showed in (N), *n* = 6 mice. O,P) Western blot analysis of BACE1 and Nicastrin expression in cortex and hippocampus of 6‐month‐old 5×FAD mice and 5×FAD+2,5‐AM mice. Quantification of relative protein levels are shown in in (P), *n* = 4 mice. Mouse number used in behavior tests: 5×FAD mice: *n* = 15 mice, 5×FAD+2,5‐AM mice: *n* = 15 mice; Data represent mean ± SEM, n.s.: not significant, ^*^ *p* < 0.05, ^**^ *p* < 0.01, ^***^ *p* < 0.001, unpaired t test for behavioral statistics. Other statistical applications were analyzed by one‐way ANOVA with Tukey's post hoc analysis.

Mannose enters the cell through glucose transporter 5 (Glut5).^[^ ^29^, ^30^ ^]^ To block the effects of mannose, its analogue: 2,5‐AM was used (**Figure** **4**A). To test the specificity of 2,5‐AM, Neuro‐2a (N2A) cells were treated with β‐amyloid and 2,5‐AM. The cellular levels of mannose and other monosaccharides (fructose, fucose, and galactose) which use Glut5 as a transporter were measured. As predicted, the cellular mannose level increased with β‐amyloid treatment, and 2,5‐AM administration blocks this increase (Figure 4B). We did not observe any changes in the cellular levels of fructose, fucose, or galactose, which indicates that 2,5‐AM administration did not affect the function of these other monosaccharides (Figure 4C–E). 2,5‐AM was then administrated into the brain of 6‐month‐old 5×FAD mice by intracerebroventricular (ICV) injection at 10 mg kg^−1^ for one dose. These mice were subjected to behavioral tests and brain slice immunostaining 48 h later (Figure 4F). Notably, the T maze (Figure 4G), Y maze (Figure 4H), and water maze tests (Figure 4I–L) indicated that the cognitive impairments of 5×FAD mice were significantly attenuated by 2,5‐AM treatments. The motor activity of AD mice treated with 2,5‐AM was not affected, as tested by the open field, rotarod, and high plus maze test (Figure S5A–D, Supporting Information). Consistent with the behavioral data, the 5×FAD mice treated with 2,5‐AM also showed a remarkable reduction of Aβ deposition (Figure 4M,N), accompanied by the decreased protein levels of BACE1 and Nicastrin in the hippocampus and cortex (Figure 4O,P). We obtained similar data in CHO‐APP cells. The increased protein levels of BACE1 and Nicastrin induced by mannose were blocked by 2,5‐AM (Figure S5E,F, Supporting Information). The production of Aβ 1–42 was also decreased by 2,5‐AM treatment in CHO‐APP cells (Figure S5G, Supporting Information).

### 2.5 Single‐Nucleus RNA Sequencing and Targeted Metabolomics Analysis Reveal that N‐Glycosylation Modifications are Primarily Affected by Mannose

**Figure 5:**
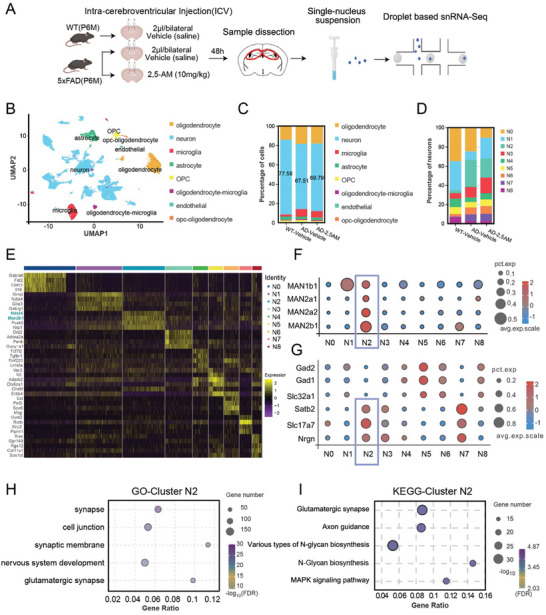
2,5‐AM affect function of neurons through N‐glycosylation modification A) Schematic of the experimental strategy used for single‐nucleus RNA sequencing from the hippocampus of 6‐month‐old wild‐type mice and 5×FAD mice treated with or without 2,5‐AM. B) UMAP representation unsupervised clustering of snRNA seq datasets of hippocampus with cells colored by cell types. C) The ratio histogram of different cell types, including neuron, oligodendrocyte, astrocyte, microglia and endothelial, etc. D) t‐SNE dimensionality reduction analysis identified 9 neuronal clusters of all neurons. E) Heatmap of the top marker genes for each neuronal cluster, Ndst4, Man2b1, Pcsk5 and Nrp1 can be regarded as marker genes for cluster N2. F) Bubble plots of expression values of main N‐Glycosylation mannosidase genes (avg.exp.scale) and the number of N‐Glycosylation mannosidase genes in 9 sub‐cluster (pct.exp). G) Bubble plots of expression values of inhibitory/excitatory neuron marker genes (avg.exp.scale) and the number of cells expressing inhibitory/excitatory neuron marker genes in 9 sub‐cluster (pct.exp). H) Functional annotation of DEGs in cluster N2 of three groups of mice by Gene ontology (GO) analysis. I) Functional enrichment of differential gene expression (DEGs) in cluster N2 of three individual samples. The plots describe the significant functionally enriched Kyoto Encyclopedia of Genes and Genomes (KEGG) pathways in cluster N2.

To gain further insights into the mechanisms underlying the mannose effect on the pathogenesis of AD, we performed single‐nucleus RNA sequencing (snRNA‐Seq) analysis of hippocampus samples from 5×FAD5×FAD and littermate control mice treated with or without 2,5‐AM (**Figure** **5**A). The detailed experimental procedures can be found in the methods section. The uniform manifold approximation and projection (UMAP) was used to reduce the dimensionality of the snRNA‐seq data to ≈ plots and identified 8 cell populations (Figure 5B). Among these cell populations, the neuron population was significantly affected by 2,5‐AM treatment. The neuronal cell population occupied 77.58% of the hippocampus in WT mice, while it decreased to 67.51% in AD mice which was increased to 69.79% by 2,5‐AM administration (Figure 5C). We next performed t‐distributed stochastic neighbor embedding (t‐SNE) and identified 9 major neuronal clusters. Among these neuronal cell clusters, the number of cells in cluster N2 increased in 5×FAD mice compared with WT mice and decreased after intraventricular injection of 2,5‐AM (Figure 5D). Two core genes for cluster N2 (*Ndst4, Man2b1*) are closely related to N‐glycosylation (Figure 5E). Bubble plots further illustrate that all N‐Glycosylation mannosidase genes were enriched in cluster N2 (Figure 5F). Further analysis using excitatory and inhibitory neuron markers revealed that cluster N2 is predominantly composed of excitatory neurons (Figure 5G).

To investigate the function of cluster N2, Gene Ontology (GO) enrichment analysis in cluster N2 revealed that the neuronal synaptic functions were highly enriched (Figure 5H). Moreover, the Kyoto Encyclopedia of Genes and Genomes (KEGG) analysis also indicated that the glutamatergic synapse and N‐glycosaminoglycan biosynthesis pathway were enriched in cluster N2 (Figure 5I). The snRNA sequencing data indicated that mannose may affect neuronal function through N‐glycosylation modification.

To elucidate detailed changes in metabolomics, the hippocampus of 6‐month‐old wildtype mice with a standard diet, 5×FAD mice with a standard diet, 5×FAD mice with mannose‐free diet, and 5×FAD mice with a mannose‐free diet+10% mannose were subjected to targeted metabolomics. We screened more than 60 major metabolites (Supplementary Dataset 1) and performed these metabolomics to metabolic reconstruction (METARECON)^[^ ^31^, ^32^ ^]^ analysis. As predicted, the glycosylation pathway was highlighted in the hippocampus of 5×FAD feeding with a mannose‐free diet (Figure S6, Supporting Information).

One major function of mannose is to participate in glycosylation by regulating glycolysis (Figure S7A, Supporting Information).^[^ ^33^ ^]^ Moreover, the major metabolites (M‐6‐P, F‐6‐P, FBP, M‐1‐P) of the glycosylation pathway increased significantly in AD mice compared to wild‐type mice. These upregulations in AD mice were blocked by feeding a mannose‐free diet and the reintroduction of mannose increased the levels of these metabolites (Figure S7B, Supporting Information). These results further confirmed that mannose free feeding disrupts glycosylation in AD mice.

### 2.6 Mannose Promotes the N‐Glycosylation and Protein Stability of BACE1 and Nicastrin

**Figure 6:**
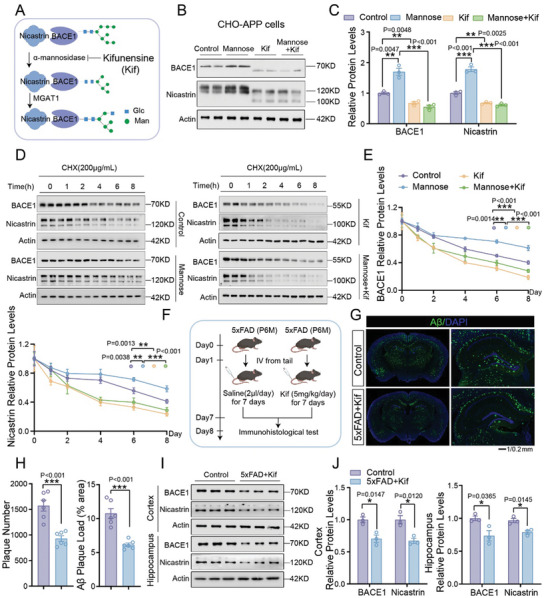
Mannose based glycosylation promote β, γ‐secretase subunits BACE1 and Nicastrin stability, aggravating the pathological process of AD. A) An overview of major steps of N‐glycan of BACE1 and Nicastrin. B, C) CHO‐APP cells were chased in the presence of Kifunensine. Western blot analysis of BACE1 and Nicastrin expression in CHO‐APP cells simultaneously treated with or without mannose (25 mM) in the presence or absence of Kifunensine (Kif). Quantification of relative proteins levels are showed in (C), *n* = 3 experimental replication/group. D,E) CHO‐APP cells were pre‐treated with vehicle control, mannose, Kif or mannose+Kif, and then were exposed to cycloheximide for the time indicated. The BACE1 and Nicastrin degradation rates were determined by western blotting. Representative blots are shown in panel (D). Quantification of proteins turnover is shown in (E), *n* = 3 experimental replication/group. F) Schematic diagram of giving Kif (5 mg kg^−1^) or control vehicle (saline) IV from the tail vein on 6‐month‐old 5×FAD mice. G, H) Immunofluorescence staining of Aβ in cerebrum from 6‐month‐old 5×FAD mice and 5×FAD+Kif mice. Representative confocal images are shown on panel (G), Scale bar:1 mm, 0 .2 mm. Quantitation of plaque number and load are showed in (H), *n* = 6 mice. I,J) Western blot analysis of BACE1 and Nicastrin expression in cortex and hippocampus of 6‐month‐old 5×FAD mice and 5×FAD+Kif mice. Quantification of relative protein levels are shown in in (J), *n* = 3 mice. Data represent mean ± SEM, n.s.: not significant, ^*^ *p* < 0.05, ^**^ *p* < 0.01, ^***^ *p* < 0.001, unpaired t test for behavioral statistics. Other statistical applications were analyzed by one‐way ANOVA with Tukey's post hoc analysis.

Protein N‐glycosylation is characterized by the attachment of oligosaccharides on asparagine residues.^[^ ^34^ ^]^ Our snRNA‐Seq and METARECON analysis indicate that mannose is highly involved in protein N‐glycosylation in AD. Accumulating evidence also suggested that the N‐glycosylation of β‐and γ‐secretases play important roles in AD pathogenesis. To further investigate the effects of glycosylation mediated by mannose in the pathogenesis of AD, Kifunensine (inhibitor of α‐mannosidase I) was used to block the N‐glycosylation synthesis (**Figure** **6**A).^[^ ^35^ ^]^ We found that Kifunensine (Kif) significantly blocks the glycosylation modification of BACE1 and Nicastrin (evidenced by a shift in the bands) and decreases their protein levels in CHO‐APP cells (Figure 6B,C). These data suggested that the glycosylation of BACE1 and Nicastrin may influence their protein stability. To test this hypothesis, Cycloheximide (CHX) was used to block the protein synthesis in CHO cells treated with either mannose or Kif. Notably, mannose administration significantly increased the protein stability of BACE1 and Nicastrin, which was blocked by Kif (Figure 6D,E). Kif was then administered to 6‐month‐old 5×FAD mice via tail vein injection for 7 days (Figure 6F). These mice were then sacrificed and the brain slices were subjected to Aβ staining. Kif administration significantly reduced the amyloid plaque burden (Figure 6G,H) accompanied by decreased protein levels of BACE1 and Nicastrin in the hippocampus and cortex of 5×FAD mice (Figure 6I,J). These results further indicate that mannose not only promotes N‐glycosylation but also enhances the stability of BACE1 and Nicastrin proteins.

## 3 Discussion

Mannose, a naturally occurring bioactive monosaccharide, plays vital roles in living organisms, participating in both metabolism and glycoprotein synthesis.^[^ ^36^ ^]^ However, our study suggests that excessively high circulating mannose levels in human subjects may indicate an increased risk of AD. Further investigation at the molecular and cellular level revealed that mannose may influence neuronal function through alterations in N‐glycosylation of BACE1 and Nicastrin, which can promote the production of Aβ plaques.

Five common monosaccharides (D‐glucose, D‐mannose, D‐fructose, L‐fucose, and L‐galactose) have gained popularity as food additives and nutritional supplements. Extensive research has established a link between glucose metabolism dysfunction and AD. Insulin resistance and impaired glucose metabolism, often associated with obesity and type 2 diabetes, are risk factors for AD.^[^ ^1^, ^37^ ^]^ Cerebral glucose metabolism dysfunction is not simply a consequence of metabolic and neurological diseases but a key factor, potentially an early event, in the pathological cascade leading to AD.^[^ ^38^ ^]^ Given that the monosaccharides all can readily pass through the blood‐brain barrier,^[^ ^39^, ^40^ ^]^ we investigated the potential effects of their levels on AD. Except for glucose, we found that only mannose is elevated in AD mice and patients, suggesting that the detection of mannose levels may play an important role in the early diagnosis of AD. Mannose exhibits the most pronounced alterations compared to other monosaccharides in AD, potentially due to its higher glycosylation rates and abundance. Glycation, which plays a significant role in the cytotoxicity of neural cells, may also contribute to these alterations.

Aβ production and clearance are pivotal for the onset of AD. Many proteins involved in Aβ production can be N‐glycosylated.^[^ ^41^, ^42^ ^]^ β‐amyloid fragment is cleaved by BACE1 from APP on the luminal side of acidic organelles, such as endosomes and the trans‐Golgi network.^[^ ^43^ ^]^ The proteins located in the luminal side or outside of the cell membrane are normally decorated with N‐ and O‐glycans. Acutely, Amyloid precursor protein (APP), BACE1,^[^ ^44^ ^]^ Nicastrin (a γ‐secretase subunit),^[^ ^45^ ^]^ ADAM10 (a protease), Neprilysin (an Aβ‐degrading enzyme), and TREM2 (triggering receptor expressed on myeloid cells 2) are all known to be N‐glycosylated.^[^ ^46^, ^47^ ^]^ Glycosylation determines the secretion, processing, and hydrolysis pathways of APP. In several experiments in which targeted mutagenesis was used to delete two potential N‐linked glycosylation sites of APP (N467 and N496), there was significantly slower initial APP secretion.^[^ ^48^ ^]^ In addition, the R47H variant of TREM2 exhibits increased terminal N‐glycosylation with complex oligosaccharides in the Golgi, leading to decreased solubility, and affecting ligand binding and receptor function.^[^ ^49^ ^]^ However, the effects of glycosylation on these proteins remain unclear.

Ideas on the inhibition of Aβ protein production through the alteration of glycosylation have mainly focused on the modulation of enzymes that process APP, such as using the curcumin derivative GT863 to inhibit N‐glycosylation of γ‐secretase.^[^ ^50^ ^]^ BACE1 can be N‐glycosylated at four potential sites, which directly correlates with its folding, secretion rates, and enzyme activity. Selective modulation of BACE1 cleavage activity toward APP by altering the glycosylation of BACE1 is considered a promising AD therapeutic modality.^[^ ^51^ ^]^ However, previous inhibitors designed to target BACE1 activity often interfere with its processing of other substrates, leading to severe side effects. BACE1 is also highly modified with N‐acetylglucosamine (GlcNAc). In vivo studies in mice have shown that knocking out the Mgat3 gene, which encodes the N‐acetylglucosaminyltransferase III (GnT‐III) enzyme, resulting in a shift in BACE1 localization toward late endosomes/lysosomes, indirectly targeting the BACE1 activity and reducing Aβ load.^[^ ^52^ ^]^ While research elucidating the role of BACE1 glycosylation in AD has gained momentum, most approaches still target BACE1 indirectly. Identifying direct modulators of BACE1 glycosylation with minimal side effects, such as those affecting protein stability, remains a challenge.

Our snRNA‐seq data indicate that N‐glycosylation of proteins is increased in neurons of 5×FAD mice and decreased after 2,5‐AM treatment. These neurons associated with glycosylation modifications were primarily excitatory neurons. Further results indicated that mannose administration significantly increased the protein stability of BACE1 and Nicastrin. These findings strength the hypothesis that mannose may affect the function of excitatory neurons through N‐glycosylation modifications, thereby exacerbating the pathology of AD. Moreover, our study revealed that mannose significantly impacted neuronal function, while no substantial alterations were observed in microglia or astrocytes. We hypothesize that this differential sensitivity may arise from the heightened susceptibility of neurons to N‐glycosylation changes. This hypothesis is supported by existing literature, which indicates that high N‐glycan diversity is essential for neuronal adhesion and renders developing cerebella vulnerable to N‐glycosylation defects.^[^ ^53^ ^]^ The abnormally N‐glycosylated proteins in AD could serve as biomarkers for clinical diagnosis and potential therapeutic targets, offering an opportunity to translate N‐glycosylation research into clinical applications.

D‐mannose, a monosaccharide naturally found in various fruits, including cranberries, blueberries, peaches, and apples, is widely present in dietary sources, offering multiple avenues for potential health benefits and adverse effects associated with daily mannose intake. Additionally, mannose has been proposed to play a beneficial role in the treatment of various diseases, including cancers and inflammatory diseases.^[^ ^11^ ^]^ However, the spatial memory ability and neuronal function of mannose‐treated mice were impaired, particularly in those treated with higher concentrations of mannose in our study. No prior studies have reported detrimental effects of mannose on cognitive function.

The 5×FAD model develops progressive Alzheimer's disease‐like pathology, with amyloid plaque deposition beginning as early as 1.5 months and reaching substantial levels by 6 months. At 6 months, these mice also show significant behavioral deficits, making this age particularly relevant for assessing treatment effects on both pathology and behavior. By selecting 6‐month‐old mice for our experiments, we ensured that the disease was well‐established, allowing us to evaluate the potential impact of mannose, 2,5‐AM, and kif on prominent AD‐like symptoms. Additionally, starting long‐term treatments at 4 months allows us to investigate preventative or early intervention effects, with assessments conducted at 6 months to capture both early and progressive changes in the disease state.

Through repeated experiments, we consistently observed a significant increase in Aβ levels in the brains of 5×FAD mice within 48 h following ICV administration of mannose. This rapid response was indeed striking, underscoring the pronounced impact of mannose on AD pathology. To our knowledge, our study is the first to investigate the impact of mannose on AD‐related pathology in 5×FAD mice models. A previous study examining the effects of mannose on cognition was conducted in wild‐type mice,^[^ ^12^ ^]^ with little exploration of its influence on amyloid dynamics in AD models. Given the limited literature on this topic, we lack direct references regarding the rapid increase in amyloid plaque deposition within 48 h following mannose administration. We hypothesize that the observed increase in amyloid plaque burden could be due to mannose accelerating Aβ production through mechanisms related to glycosylation modifications. Specifically, mannose may alter the glycosylation of key proteins involved in Aβ processing, such as BACE1 and Nicastrin, both of which are crucial for amyloid precursor protein (APP) cleavage and subsequent Aβ generation. Enhanced glycosylation of these enzymes could potentially drive an abnormal increase in Aβ production over a relatively short time frame. Additionally, our single‐cell RNA sequencing data suggest that mannose may also impact neuronal function, further contributing to rapid amyloid deposition. Further research is needed to clarify the specific mechanisms involved.

Although a single administration of mannose into the brain does not replicate lifelong high mannose consumption, our experiments surprisingly revealed that even a single ICV injection of mannose can exacerbate plaque deposition in the brains of 5×FAD mice (Figure 2B), demonstrating a close relationship between mannose metabolism and disease progression. Additionally, this experimental design also aims to evaluate the direct impact of mannose on the brain while minimizing the influence of peripheral metabolism. These findings may establish a foundation for understanding the potential direct effects of mannose on AD pathology and offer guidance on the potential benefits of restricting mannose intake for improving the pathological progression of AD. While our investigation delved into the effects of mannose on the cortex and hippocampus, the influence of mannose on other brain regions merits further investigation. Given the prevalence of mannose in daily life and its diverse clinical applications, our study raises significant concerns regarding the potential deleterious effects of chronic mannose intake on the nervous system and its role in AD.

We conducted additional experiments in which wild‐type mice were given 20% mannose‐supplemented water for a period of two months, finding that such supplementation led to notable cognitive impairment in these animals. This observation is consistent with prior studies,^[^ ^12^ ^]^ which demonstrated that high‐dose mannose specifically impairs spatial memory in the Morris water maze without affecting spatial learning in wild‐type mice. Based on these findings, we propose that prolonged exposure to high concentrations of mannose may contribute to cognitive decline in wild‐type mice.

In this study, we first observed that intracerebroventricular (ICV) injection of mannose (60 mg kg^−1^) in 5×FAD mice resulted in a significant increase in Aβ plaque burden, indicating that high doses of mannose can exacerbate Alzheimer's disease (AD) pathology. Subsequently, long‐term administration of 20% mannose in drinking water for 60 days led to cognitive decline and aggravated pathology in 5×FAD mice. Importantly, this detrimental effect was alleviated by either a mannose‐free diet or co‐administration of mannose antagonists (2,5‐AM or kifunensine).

Mechanistically, metabolomic and single‐cell RNA sequencing analyses revealed that mannose influences Aβ production by modulating the glycosylation of key amyloidogenic proteins, including BACE1 and Nicastrin. These findings highlight the potential risks associated with high‐dose and prolonged mannose exposure, as such conditions exacerbate AD pathology and cognitive impairment.

While previous studies have highlighted the therapeutic potential of mannose in oncology,^[^ ^10^, ^54^, ^55^ ^]^ our results provide critical insights into the complex role of mannose in AD progression and raise concerns regarding its long‐term supplementation, especially at high doses, in both healthy individuals and populations at risk for neurocognitive disorders. Future studies are warranted to further explore the dose‐response relationship and the long‐term neurocognitive effects of mannose under different physiological and pathological conditions.

In summary, we found that mannose levels are elevated in both AD mice and patients. Mannose‐mediated glycosylation stabilized BACE1 and Nicastrin, promoting the generation of β‐amyloid. While mannose has demonstrated several benefits in anti‐tumor functions, prolonged high‐dose exposure may impair cognition and exacerbate the pathogenesis of AD.

## 4 Experimental Section

### Patient and Control Samples

Serum samples from AD patients and controls were acquired from Huashan Hospital, affiliated with the Shanghai Medical College of Fudan University, Shanghai, P.R. China, and the Department of Health Examination, the First Affiliated Hospital of Xiamen University. Study protocols were approved by the Ethics Committee of the First Affiliated Hospital of Xiamen University. The approval number of the protocol was XDYX202101K11. Informed content was written and obtained from all subjects. All samples were collected in adherence with the Declaration of Helsinki. Participant characteristics are provided in **Table** **1**.

**Table 1:** Characteristic information of AD patients and controls in this study.

	Case	Control (>60)
Number of subjects	8	8
Sex (male/female)	5/3	4/4
Age (years)	Male: 59.50 ± 1.50 Female: 64.57±1.185	Male: 70.08±1.695 Female: 68.98±1.128
Age of onset (years)	56.34±1.286	N/A

### Animals

All mice were maintained within the core animal facility at Xiamen University, and all experimental procedures involved were conducted according to protocols approved by the Institutional Animal Care and Use Committee at Xiamen University. The approval number of the protocol was XMULAC20200054. Mice were housed under a 12‐h light/dark cycle with free access to standard rodent chow and water. Each cage contained a maximum of four mice. Mice were maintained under specific‐pathogen‐free (SPF) conditions and were not subject to immune suppression. The health of the animals used was regularly controlled by animal caretakers. All mice used were drug/test naïve. The ambient temperature in the facility was at 20–25 °C, with 40–60% humidity. 5×FAD mice were purchased from Jackson Lab (B6. Cg‐Tg (APPSwFlLon, PSEN1*M146L*L286 V) 6799Vas/Mmjax, JAX34848). The experimental mice were generated by crossing 5×FAD mice with C57BL/6 wild‐type mice, ensuring the use of littermate controls. All breeding, housing, and treatments (e.g., mannose supplementation) were conducted at the Xiamen University Animal Center. Prior to behavioral testing, mice were transferred to the behavioral testing room and acclimated for at least 24 h to minimize stress and ensure consistent results. A mixture of 3‐, 4‐, 5‐, 6‐, and 7‐month‐old litter/age‐matched mice were employed in all studies. Additionally, a mixture of 4‐month‐old litter/age‐matched wildtype mice were employed in feeding 20% mannose study. All animals were used according to the “3Rs” principles (Replacement, Reduction, and Refinement) in experimental procedures.

Previous literature has demonstrated that 5×FAD mice exhibit significant behavioral differences as early as 6 months of age.^[^ ^56^ ^]^ Regarding the choice of specific ages and treatment durations, in our study, 6‐month‐old 5×FAD and littermate control mice were used for the experiments involving injections of mannose, 2,5‐AM, or kif, as 5×FAD mice generally exhibit prominent behavioral deficits and pathological features around this age. For long‐term drinking water experiments, the study began with 4‐month‐old AD and control mice, allowing for a two‐month treatment period so that behavioral assessments could be conducted at 6 months of age when AD‐related characteristics were more pronounced.

Furthermore, wildtype mice were used as the control group for the 5×FAD model, and both groups were treated under the same experimental conditions, including feeding mannose and administration of 2,5‐AM. For the mannose supplementation experiments, three approaches were implemented. The first approach entailed stereotactic injection of mannose into the lateral ventricle of 6‐month‐old 5×FAD mice, with a control group of 5×FAD mice receiving equivalent volumes of solvent. Tissue samples were collected 48 h post‐injection. The second approach involved feeding 4‐month‐old wildtype and 5×FAD mice with either standard water or 20% mannose‐enriched water for a duration of two months (wildtype group data are presented in Figure S3, Supporting Information). For the third approach, 4‐month‐old wildtype and 5×FAD mice were divided into four distinct groups: wildtype and 5×FAD mice on a standard diet, 5×FAD mice on a mannose‐free diet (AIN‐93G Growth Purified Diet, Testdiet, #5801‐G), and 5×FAD mice on a mannose‐free diet with 10% mannose in the drinking water. This regimen was maintained for 70 days, with all groups undergoing behavioral testing at 6 months of age.

The volume of water supplemented with mannose was calculated based on the frequency of water changes during the treatment period, along with the total volume of each water bottle used. Mice were weighed twice: once prior to the commencement of the mannose treatment and again immediately following the completion of the treatment, just before behavioral testing.

### Experimental Design

All experiments described in this study were performed with a minimum of 3 mice or 3 independent experiments. The sample size per experiment was determined according to previous publications.^[^ ^57^ ^]^ To determine the required sample size for behavioral experiments, a power analysis was also conducted using G*Power software. Preliminary data indicated an expected mean difference of ≈20 between groups, with a standard deviation of 10, resulting in an estimated effect size of 2.0. For this calculation, the significance level (α) was set at 0.05 and the statistical power at 0.8, which yielded a required sample size of 10–15 mice per group. This sample size ensures sufficient power to detect significant differences between experimental conditions. All in vitro experiments described in this study were performed with a minimum of 3 independent biological replicates (different samples) to ensure reproducibility and accuracy. For example, in our western blot experiments using cell samples, multiple samples within each group were prepared from different plates/flasks under identical conditions (at least 3 independent experiments). For western blot and immunofluorescence staining using tissue samples, each group consisted of samples collected from at least three different mice, providing biological replicates. Additionally, each sample was measured in technical triplicate within each Western blot experiment. Statistical analysis was then performed on these technical and biological replicates to ensure the robustness of the data. All sample selections were randomized and unbiased, with all statistical analyses conducted in a blinded manner to prevent any potential bias in data interpretation. Specifically, the mouse genotype was de‐identified during the experimental trials in the behavioral analyses described.

### Drug Preparation and Administration

For in vivo experiments, D‐mannose (Sigma‐Aldrich, M8574), 2,5‐anhydro‐D‐mannitol (2,5‐AM) (MACKLIN, A888642), and Kifunensine (Kif) (MCE, FR‐900494) were dissolved in Stroke‐Physiological Saline Solution (SPSS). Kifunensine was administered intravenously (IV) via the tail vein at a dose of 5 mg kg^−1^ once daily for 7 days.^[^ ^58^ ^]^ For intracerebroventricular (ICV) injections, 6‐month‐old 5×FAD mice were anesthetized and positioned in a stereotaxic apparatus. After making a small skin incision, holes were drilled at coordinates targeting the lateral ventricle (±1.0 mm lateral, −0.4 mm anterior‐posterior from bregma). D‐mannose and 2,5‐AM were administered ICV at 60 and 10 mg kg^−1^, respectively.^[^ ^59^, ^60^, ^61^ ^]^ Each injection (total volume of 2 µl for D‐mannose or 2 µl for 2,5‐AM) was delivered at a rate of 0.2 µl min^−1^ to a depth of 2.2 mm. The injection syringe was kept in place for an additional 10 min to ensure proper diffusion before being carefully withdrawn. For in vitro experiments, CHO‐APP or N2A cells were treated with the following compounds dissolved in the cell culture medium at indicated final concentrations: D‐mannose at 25 mM, Kifunensine at 10 µM, 2,5‐AM at 600 µM, and Cycloheximide (CHX) (MCE, HY‐12320) at 710 µM.

For D‐mannose (Sigma–Aldrich, M8574), It was administered ICV at 60 mg kg^−1^ or 20% mannose in drinking water for the in vivo studies and at 25 mm for the in vitro studies. This dosage were supported by Gonzalez et al., who demonstrated that mannose can impair tumor growth and enhance chemotherapy.^[^ ^62^ ^]^ To explore the effect of mannose on tumors, they treated Saos‐2, U2OS‐E1a, and KP‐4 cells with 25 mm mannose for the in vitro studies and treated C57/BL6J wild‐type mice with 20% mannose in drinking water. In addition, no one has injected mannose directly into the brain, but Xu et al. reported low‐dose MAN (0.48g kg^−1^) or high‐dose MAN (4.8g kg^−1^) intraperitoneally injected (i.p.) for one month resulted in spatial memory impairment in wildtype mice.^[^ ^12^ ^]^ The ICV injection concentration of mannose at 60 mg kg^−1^ was chosen based on this dosage.

Kifunensine (Kif) (MCE, FR‐900494) was administered intravenously (IV) via the tail vein at 5 mg kg^−1^ for seven days in vivo and at 10 µm for in vitro studies. This dosage is in accordance with findings from Choi et al., who utilized N‐glycan remodeling in transgenic rice cell cultures.^[^ ^22^ ^]^

For 2,5‐AM (MACKLIN, A888642), it was administered ICV at 10 mg kg^−1^ for in vivo studies and at 600 µM for in vitro studies. This was consistent with Allison et al., who examined the effects of common nitrosating agents on chitosan using a glucosamine oligosaccharide model system.^[^ ^24^ ^]^

### N2A Cells and CHO‐APP Cells Culture Procedures

N2A cells and CHO‐APP cells were purchased directly from ATCC, and have been authenticated by ATCC. N2A cells and CHO‐APP cells were utilized in passage 4, and cultured in DMEM (Dulbecco's Modified Eagle Medium) supplemented with 10% fetal bovine serum (FBS) under standard conditions (37 °C, 5% CO₂, humidified atmosphere). CHO cell line stands for Chinese Hamster Ovary cell line, characterized by its ability to stably integrate foreign genes, enabling efficient amplification and expression. The human APP‐PS1 double‐gene transfected CHO cell line (CHO‐APP cell line, 7WPS1), was created by using the liposome transfection method to transfer the wild‐type PS1 gene into CHO cells that stably express the APP gene. CHO‐APP cells were selected specifically due to their stable overexpression of human amyloid precursor protein (APP), which facilitates consistent production and secretion of Aβ. This characteristic makes CHO‐APP cells particularly valuable for investigating how mannose affects the stability of key proteins like BACE1 and Nicastrin, both essential in APP processing and Aβ production. CHO‐APP cells were treated with 25 mM D‐Mannose or 10 µm Kifunensine and 200 µg mL^−1^ CHX for 1, 2, 4, 6, and 8 h to assess BACE1 and Nicastrin stability. N2A cells were treated with 1 µm Aβ₁₋₄₂ and 600 µm 2,5‐AM for 48 h to assess intracellular mannose levels.

Treatment paradigms for cell culture experiments: For experiments shown in Figure 4B using N2A cells: Cells were divided into three groups and treated simultaneously with i) vehicle control, ii) Aβ oligomers, or iii) Aβ oligomers and 2,5‐AM (overlapping treatment). Samples were collected 48 h after treatment. For experiments shown in Figure 6B using CHO‐APP cells: Cells were divided into four groups and treated simultaneously with i) vehicle control, ii) mannose, iii) kifunensine (Kif), or iv) mannose and Kif (overlapping treatment). Samples were collected 48 h after treatment. For experiments shown in Figure 6D, CHO‐APP cells from the four groups (control, mannose, Kif, and mannose + Kif) were subjected to six additional time gradients of cycloheximide (CHX) exposure (0, 1, 2, 4, 6, and 8 h). These experiments resulted in 24 groups in total, with CHX added at 40, 42, 44, 46, and 47 h after the initiation of the respective treatments.

### Behavioral Studies

As our main objective in conducting behavioral experiments was to investigate neurofunctional aspects related to learning and memory, part of the experiments was limited to behavioral tasks specifically associated with learning and memory (Figure 2D–G; Figure S3D–I, Supporting Information). The sequence of these behavior tests is as follows: T‐maze on day 1, followed by a 2‐day rest period. Y‐maze on day 4, followed by a 3‐day rest period. Morris water maze training on days 8–13, and the Morris water maze test on day 14. For Figure 3B–E and Figure S4 (Supporting Information), a comprehensive suite of behavioral assessments was conducted. The behavior tests were conducted over a period of 21 days as follows: Open field test on day 1, followed by a 1‐day rest period. T‐maze on day 3, followed by a 1‐day rest period. Y‐maze on day 5, followed by a 1‐day rest period. Morris water maze training on days 7–12, and the Morris water maze test on day 13, followed by a 1‐day rest period. On day 14, the High plus maze was conducted, followed by a 1‐day rest period. Rotarod test on day 17, followed by a 1‐day rest period. Tail suspension test on day 19, followed by a 1‐day rest period, and Forced swim test on day 21. For Figure 4G–L and Figure S5 (Supporting Information), the behavior tests were conducted over a period of 17 days as follows: Open field test on day 1, followed by a 1‐day rest period. T‐maze on day 3, followed by a 1‐day rest period. Y‐maze on day 5, followed by a 1‐day rest period. Morris water maze training on days 7–12, and the Morris water maze test on day 13, followed by a 1‐day rest period. On day 15, the High plus maze was conducted, followed by a 1‐day rest period, and the Rotarod test on day 17. The recommended interval between two behavioral tasks varies widely across different literature. According to this particular literature,^[^ ^62^ ^]^ there was no significant difference in behavioral outcomes between groups with an interval of 1–2 days and those with an interval of 1 week. Therefore, a 48 h interval was implemented between the two behavioral tasks to reduce stress and fatigue effects on the mice. Each test was performed on a separate day to minimize potential interference, allowing at least 48 h between tests to reduce stress and fatigue effects on the mice.

T and Y maze: The apparatus comprised three closed arms (50 × 10 cm) which were formed T or Y type. Connected by a central square platform and positioned 50 cm above the ground. Mice were placed in the middle of the platform, and their behavior was tracked for 10 min with an overhead camera and Smart 3.0 software. Attention triplet was reported.

### Forced Swimming Test

The forced swim test (FST) was used to assess depressive‐like behavior. Mice were placed in a container filled with water that eventually resulted in immobility, reflecting behavioral despair. Water (23 ± 1 °C) was placed in a transparent acrylic cylinder bath (10cm in diameter, 20 cm in height) filled to a depth of 13cm. Mice were exposed to the water for 6 min using a video tracking system (Smart 3.0); immobility duration (%) within the final 5 min of testing was recorded.

### Tail Suspension Test

The tail‐suspension test was used to assess the efficacy of antidepressants in mice. Mice were suspended by their tails from an acrylic bar (15cm in diameter, 30 cm in height) for 6 min and monitored using a video tracking system (Smart 3.0). Escape‐related behavior was assessed, where immobility duration (%) during the 6 min suspension period was recorded.

### Rotarod Test

Mice were placed on a stationary rotarod (AccuRotor Rota Rod Tall Unit, 63 cm fall height, 30 mm diameter rotating dowel; Accuscan, Columbus, OH). The dowel was then accelerated to 60 rpm min^−1^, and the latency to fall (in seconds) was recorded. The procedure was repeated over 4 consecutive trials, which was averaged to yield the daily latency to fall for each mouse. If an animal fell off the rotarod rapidly (e.g., due to inattention or slips), they were placed back on the rotarod for an additional trial, and the latency was not included in the daily average. The entire procedure was repeated for 2 additional days for a total of 3 days. In addition to the average latency across the 4 trials per day, the maximum latency to fall per day was also analyzed.

### Open Field Test

To explore locomotion and spontaneous activity, mouse behavior was characterized as they freely explored an open‐field plastic chamber (40 cm width × 40 cm length × 30 cm height) using a video tracking system (Smart 3.0). 6‐month‐old mice were placed in this arena for 10 min, and total distance and time spent in the center region (20cm × 20 cm) were recorded.

### High Plus Maze

The apparatus comprised two opposing open arms (50 × 10 cm) and two opposing closed arms with roofless gray walls (40 cm) connected by a central square platform and positioned 50 cm above the ground. Mice were placed in the open arms facing an open arm, and their behavior was tracked for 5 min with an overhead camera and Smart 3.0 software. Time spent in the open arms (%) was reported.

### Morris Water Maze Behavior Test

The water maze used in this study comprised a circular tank 120 cm in diameter with a platform filled with tap water at a temperature of 22 ± 2 °C. Different shapes were posted along the walls of the tank, which served as spatial reference cues. A camera was mounted above the maze to record the swimming traces in the water maze. During the acquisition trials, the platform was submerged 1–2 cm below the water surface, and mice were placed into the maze at one of four points (N, S, E, W) facing the wall of the tank. Mice were allowed to search for the platform for up to 60s. If a mouse failed to find the platform, it was guided to the platform and maintained on the platform for 10s. Four trials a day were conducted with an intermission of 1 h minimum between trials. Escape latency indicative of spatial memory acquisition, was recorded for each trial. On day 7, the platform was removed and a probe test was conducted. The percentage time spent in each of the four quadrants and the number of target (platform) area crossings, mean speed, and total distance were recorded.

### Single‐Nucleus RNA Sequencing (snRNA‐Seq) Methodology

To generate snRNA‐Seq data, hippocampal tissues were collected from three groups of 6‐month‐old mice following bilateral intracerebroventricular injection of 2,5‐AM or control vehicle (for WT and 5×FAD mice). Tissues were processed and sequenced by Genedenovo Co., using the 10 x Genomics platform for single‐nucleus capture and library preparation. Sequencing was conducted on an Illumina platform. Raw sequencing data were pre‐processed and clustered using the Cell Ranger software (v3.1) and analyzed in R with Seurat (v3.0) for quality control and downstream analysis. Following alignment to the GRCh38 genome (including intronic and exonic regions), nuclei were identified and filtered to exclude low‐quality or doublet nuclei. Specifically, nuclei expressing between 200 to 6000 genes were retained, with fewer than 5% of reads mapped to mitochondrial genes, yielding an average of 14393 high‐quality nuclei per sample. Gene expression normalization was performed using Seurat's LogNormalize function, scaling the data with a factor of 10 000. Highly variable genes were identified (*n* = 2000), and dimensionality reduction was conducted with PCA for the top 30 principal components. the FindClusters function (resolutio*n* = 0.5)was applied, and UMAP for 2D visualization of clusters (using the top 15 PCs). Cell‐type identification for clusters relied on AUCell scoring against markers from previous human brain snRNA‐Seq studies and was visually validated with marker gene expression. Cluster‐specific genes were identified with FindMarkers, and differential expression analysis between samples was conducted. Pathway enrichment for differentially expressed genes (DEGs) was performed using the KEGG database, with significance thresholds set to a fold‐change ≥2 and FDR ≤0.001. Enrichment was validated using the EWCE (weighted cell‐type enrichment) algorithm in LIGER for integrative clustering (with *k* = 20, λ = 5), providing additional insights into cell‐type distribution across experimental conditions.

### ELISA

Mannose levels in human serum, Aβ1‐42, mannose, glucose, fucose, galactose, and fructose levels in mouse serum and hippocampus/cortex were measured using ELISA Assay Kits following the manufacturer's protocols. Kits used include the Mouse Aβ1‐42 ELISA Kit (AMEKO, CAT 202004), Mannose ELISA Kit (Shanghai EYKITS, CAT CS‐0402), Glucose ELISA Kit (Shanghai Rongsheng Biotech Co., Ltd., CAT 361510), Fructose ELISA Kit (Beijing Biolab Co., Ltd., CAT SK147‐2), Fucose ELISA Kit (Megazyme, CAT.NO.K‐FUCOSE), and Galactose ELISA Kit (Henghuibio, CAT 28639BS2201).

### Oligo‐Aβ42 Preparation

Unlabelled Aβ42 (AS‐20276) was from AnaSpec. The preparation of oligomeric Aβ42 (oligo‐Aβ42) was carried out as follows: Initially, the Aβ42 peptides were dissolved in dimethyl sulfoxide (DMSO) to achieve a concentration of 5 mm. This solution was then diluted in cold phenol‐free F‐12 cell culture media (Gibco) to a final concentration of 100 µm. The diluted peptide solution was sonicated for 10 min to facilitate the dissolution and prevent aggregation, yielding a freshly prepared monomeric Aβ42 (mAβ42) solution. To induce oligomerization, a portion of the freshly prepared mAβ42 was incubated at room temperature (22 °C) for 16 h, followed by a further incubation at 4 °C for an additional 24 h. After incubation, the solution was centrifuged at 16 000 g for 15 min to remove insoluble aggregates. The supernatant, containing the oligomeric Aβ42, was carefully collected and stored for subsequent experiments at a final concentration of 100 nM.

### Western Blotting

Mice were euthanized by CO₂ asphyxiation, and tissues (including the hippocampus and cortex) were immediately harvested and stored at −80 °C. For protein extraction, tissues, and cultured cells were homogenized in ice‐cold RIPA lysis buffer containing protease inhibitors for 40 min. Lysates were centrifuged at 12 000 rpm for 10 min at 4 °C, and supernatants were transferred to clean tubes. Protein concentrations were determined using a BCA Protein Assay Kit (Thermo Fisher Scientific), and 30 µg of each sample was resolved by 10% SDS‐PAGE before being transferred onto PVDF membranes using electrotransfer in an ice‐cold buffer (25 mM Tris‐HCl, 192 mM glycine, and 20% methanol) for 1.5 h. After blocking, membranes were incubated overnight at 4 °C with primary antibodies specific for the target proteins, including BACE1 (rabbit, 1:1000; CST, #5606S), Nicastrin (rabbit, 1:1000; CST, #5665S), and Actin (mouse, 1:10 000; Novus, #NB600‐501H). Blots were then probed with goat anti‐mouse (#AP132P) and goat anti‐rabbit (#AP124P) secondary antibodies (Millipore) for 1 h at room temperature. Immunoblot bands were visualized using an ECL detection system, and images were captured and quantified using ImageJ. Band intensities were normalized to β‐actin and averaged across at least three independent experiments.

### Immunofluorescence Staining

For immunofluorescence, brain tissue was collected from euthanized mice and promptly fixed in 4% paraformaldehyde at 4 °C for 24 h. Fixed tissues were then cryoprotected in a 30% sucrose solution for 48 h, embedded in OCT, and stored at −80 °C until sectioning. Coronal brain sections were prepared at a thickness of 30 µm using a cryostat (Leica), and free‐floating sections were stored in PBS with 0.1% sodium azide at 4 °C until staining. Sections and cultured cells were washed three times in PBS and subjected to antigen retrieval in citrate buffer (pH 7.0) for 10 min at 95 °C. After cooling, sections were permeabilized and blocked in PBS containing 0.5% Triton X‐100 and 10% normal goat serum for 1 h at room temperature. Primary antibodies, including Aβ (MOAB‐2, mouse, 1:500; Biosensis, M‐1586‐100), were diluted in blocking buffer and applied overnight at 4 °C. Following three washes in PBS, sections were incubated with Alexa Fluor 488 donkey anti‐mouse secondary antibodies (1:500; Invitrogen) in a blocking buffer for 1 h at room temperature. After the final washes, sections were counterstained with DAPI in a mounting medium (Invitrogen) to visualize nuclei. Images were acquired using a Nikon A1R confocal microscope under identical acquisition settings for all samples. For IHC quantification, three coronal sections per mouse were analyzed, with sections spaced ≈200 µm apart to ensure consistent sampling. The number of plaques reported represents the total count within the cortical and hippocampal region of each section, not across all sections, and results were averaged per region of interest (ROI). Image analysis was conducted using ImageJ software, with appropriate thresholding applied to standardize plaque identification and reduce bias.

### Quantitative RT‐PCR

Total RNA was extracted from animal tissues and cultured cells using TRIzol reagent (Invitrogen), following the manufacturer's protocol. The RNA concentration and purity were assessed by spectrophotometry, and samples with an A260/A280 ratio between 1.8 and 2.0 were used for further analysis. Reverse transcription was carried out using the ReverTra Ace qPCR RT Master Mix (Toyobo, FSQ‐201), with 2 µg of total RNA per 20 µL reaction volume, to synthesize cDNA. Quantitative PCR was conducted using SYBR Green I Master (Roche) on a real‐time PCR system, with β‐actin serving as the internal control gene. Samples were assayed in triplicate and β‐actin was used as an internal control. Primer sequences used in this study:

**Table:** 

Gene	Primer sequences
*BACE1*	Forward 5′‐GGAACCCATCTCGGCATCC‐3′
	Reverse 5′‐TCCGATTCCTCGTCGGTCTC‐3′
*Ncstn*	Forward 5′‐TCCGTGGTACTGGCAGGATT‐3′
	Reverse 5′‐CCCCTGTATCCCCACTAATTGA‐3′
*Actin*	Forward 5′‐AGTGTGACGTTGACATCCGTA‐3′
	Reverse 5′‐GCCAGAGCAGTAATCTCCTTC‐3′

### Targeted Metabolomics

Sample Preparation: The hippocampus of wildtype mice with standard diet (WT+SD), 5×FAD mice with standard diet (AD+SD), 5×FAD mice with no‐mannose diet (AD+NMD), and 5×FAD mice with no‐mannose diet+10% mannose (AD+NMD+M) were weighed before the extraction of metabolites and dried lyophilized were ground in a 2 mL Eppendorf tube containing a 5 mm tungsten bead for 1 min at 65 Hz in a Grinding Mill. Metabolites were extracted using 1 mL precooled mixtures of methanol, acetonitrile, and water (v/v/v, 2:2:1) and then placed for 1 h ultrasonic shaking in ice baths. Subsequently, the mixture was placed at −20 °C for 1 h and centrifuged at 14 000 g for 20 min at 4 °C. The supernatants were recovered and concentrated to dryness in a vacuum. UHPLC‐MS analysis: The LC/MS portion of the platform was based on a Thermo Fisher Vanquish UHPLC equipped with an ACQUITY UPLC BEH Amide column (1.7 µm, 2.1 mm × 100 mm, Wasters) and a Thermo‐TSQ Vantage mass spectrometer. Energy metabolites were monitored in electrospray negative‐ionization and positive‐ionization mode. The 2 µL samples were injected sequentially into a Thermo‐TSQ Vantage mass spectrometer equipped with a Vanquish UHPLC system with autosampler (Thermo Fisher). The ACQUITY UPLC BEH Amide column (1.7 µm, 2.1 mm × 100 mm, Wasters) was heated to 45 °C under a flow rate of 300 µL min^−1^. A gradient was used to separate the compounds consisting of 20 mM ammonium acetate (solvent A) and 5% acetonitrile (solvent B). The gradient started at 5% solvent A for 1 min and increased linearly to 35% solvent A over 13 min, and then increased linearly to 60% solvent A over 2 min with a 2 min hold before returning the starting mixture during 0.1 min and re‐equilibrating for 4 min. QC samples were injected every six or eight samples during acquisition. The MS conditions were as follows: Collision Gas Pressure (mTorr): 1.0; Q1 Peak Width (FWHM): 0.70; Q3 Peak Width (FWHM): 0.70; Cycle Time (s): 1.500; Capillary Temperature: 350.0 °C; Vaporizer Temperature: 350.0 °C; Sheath Gas Pressure: 35.0; Aux Valve Flow: 10.0; Spray Voltage: Positive polarity −3500.0 V; Negative polarity −3000.0 V; scan type: selected reaction monitoring/multiple reaction monitoring (SRM/MRM). A summary of the typical precursor‐product ion‐pairs of targeted energy metabolites was listed in the supplementary information Table. Data preprocessing and filtering: Raw MRM data files were processed by peak finding, alignment, and filtering using Xcalibur Qual browser software. Multivariate statistical analysis: Simcap 14 software (Umetrics, Umeå, Sweden) was used for all multivariate data analyses and modeling. Data were mean‐centered using Pareto scaling. Models were built on principal component analysis (PCA), orthogonal partial least‐square discriminant analysis (PLS‐DA), and partial least‐square discriminant analysis (OPLS‐DA). All the models evaluated were tested for over fitting with methods of permutation tests. The descriptive performance of the models was determined by R2X (cumulative) (perfect model: R2X (cum) = 1) and R2Y (cumulative) (perfect model: R2Y (cum) = 1) values while their prediction performance was measured by Q2 (cumulative) (perfect model: Q2 (cum) = 1) and a permutation test (*n* = 200). The permuted model should not be able to predict classes: R2 and Q2 values at the Y‐axis intercept must be lower than those of Q2 and the R2 of the non‐permuted model. OPLS‐DA allowed the determination of discriminating metabolites using the variable importance on projection (VIP). The VIP score value indicates the contribution of a variable to the discrimination between all the classes of samples. Mathematically, these scores were calculated for each variable as a weighted sum of squares of PLS weights. The mean VIP value was one, and usually, VIP values over one were considered significant. A high score is in agreement with a strong discriminatory ability and thus constitutes a criterion for the selection of biomarkers. The discriminating metabolites were obtained using a statistically significant threshold of variable influence on projection (VIP) values obtained from the OPLS‐DA model and two‐tailed Student's t test (p value) on the normalized raw data at the univariate analysis level. The p value was calculated by one‐way analysis of variance (ANOVA) for multi‐group analysis. Metabolites with VIP values greater than 1.0 and p value less than 0.05 were deemed to be statistically significant metabolites. Fold change was calculated as the logarithm of the average mass response (area) ratio between two arbitrary classes. On the other side, the identified differential metabolites were used to perform heatmap analyses with the R package.

### Metabolic Reconstruction (METARECON) Strategy

To identify a specific metabolic pathway which affected by mannose in the hippocampus, a new method termed METARECON was applied. The functional integration of GC‐MS metabolomics data into a biochemical metabolic network structure was performed by the inverse approximation of the biochemical Jacobian matrix. This approximation directly connects the covariance matrix (COV), which was built from the experimental metabolomics data to the metabolic network structure of the primary metabolism. The metabolic network model is provided in Figure 5M. First, an appropriate simulation condition and data structure were made. The generic type of Equation (1) is known as the “Lyapunov Equation”, which was widely applied to control systems.

(1) $$JAC*COV+COV*JAC^{T}=-2FLU$$

where JAC represents the Jacobian matrix, the COV matrix is derived from the biological variance of independent replication analysis between a set of samples, and FLU is a fluctuation matrix that integrates a Gaussian noise function simulating metabolic fluctuations under steady‐state conditions. Jacobian matrix JAC is calculated from diffusion matrix FLU and covariance matrix COV constructed from metabonomic data. Equation (1)not only considers the noise in the data captured by the fluctuation matrix but also combines the statistical characteristics of the data with the dynamic characteristics of a stable system.

Then, the metabolism pathway efficiency consistent were made. In a biochemical environment, *r* represents the rates for each reaction and *C* represents metabolite concentration changes, each element of JAC means the elasticity of reaction rates to any change in metabolite concentrations. The corresponding Jacobian was a matrix of all first‐order partial derivatives of all functions *r_i_* on all metabolites *C_j_*, as shown in Equation (2) the Jacobian matrix JAC in (1) is defined as

(2) $$\mathrm{JAC}={\left[\begin{array}{ccc} \frac{\partial r_{1}}{\partial C_{1}} & \frac{\partial r_{1}}{\partial C_{2}}\cdots & \frac{\partial r_{1}}{\partial C_{n}}\\ \frac{\partial r_{2}}{\partial C_{1}} & \frac{\partial r_{2}}{\partial C_{2}}\cdots & \frac{\partial r_{2}}{\partial C_{n}}\\ \vdots & \ddots & \vdots \\ \frac{\partial r_{n}}{\partial C_{1}} & \frac{\partial r_{n}}{\partial C_{2}}\cdots & \frac{\partial r_{n}}{\partial C_{n}} \end{array}\right]}_{n\times n}$$

The next step is to consolidate the dynamic equation of the biological reaction. In this algorithm, the Jacobian matrix is used to describe the local dynamic characteristics near the steady state of the system. A set of differential equations represent the dynamics of metabolic pathways composed of multiple metabolites. As shown in Equation (3), where reaction rates *r*(*r*_1_,*r*_2_, …, *r_n_*) are the functions of metabolite concentrations *C*(*C*_1_,*C*_2_, …, *C_n_*)over time.

(3) $$r_{i}\left(C_{1},C_{2},\text{…},C_{n}\right)=\frac{dC_{i}}{dt}$$

By this means, as described in Equation (4), the matrix describes the change of each metabolite and its impact on the change of other metabolites.

(4) $${\left[\begin{array}{ccc} COV\left(C_{1},C_{1}\right) & COV\left(C_{1},C_{2}\right)\cdots & COV\left(C_{1},C_{n}\right)\\ COV\left(C_{2},C_{1}\right) & COV\left(C_{2},C_{2}\right)\cdots & COV\left(C_{2},C_{n}\right)\\ \vdots & \ddots & \vdots \\ COV\left(C_{n},C_{1}\right) & COV\left(C_{n},C_{2}\right)\cdots & COV\left(C_{n},C_{n}\right) \end{array}\right]}_{n\times n}$$

The METARECON approach links the kinetic equation of the metabolic pathway represented by Equation (3) to the covariance of the relevant metabolite concentration data represented by Equation (4). The above methods are combined to further substitute the data into Equation (2). This includes the integration of precursor synthesis, substrate utilization, and energy conversion pathways in biological metabolism. The last step was model improvement through metabolic interaction reannotation. JAC matrix has more independent variables than a symmetric covariance matrix, so a parametric solution was needed to eliminate the uncertainty. By quoting RENEW to determine the linear system, the solution of the Jacobian matrix was obtained. RENEW was a metabolic interaction matrix, also known as a stoichiometric matrix, which represents the interdependence of metabolic flux and metabolites. In fact, there exists regulation between metabolites without substance consumption. Then the non‐ zero in the Jacobian matrix were determined by importing the stoichiometric matrix of the metabolic network, and point out potential reactions in the basic biochemical network for the final analysis. Based on the above theorem, a strict transformation was given from Equation (2) to (5).

(5) $$JAC=RENEW\frac{\partial r}{\partial C}$$

### Statistical Analysis

All data presented were expressed as arithmetic mean ± SEM. Statistical analyses were performed using GraphPad Prism version 5.0. Null hypotheses were rejected at *p*‐values ≤0.05. For comparisons between the two groups, a Shapiro‐Wilk normality test (Prism) was first conducted to assess the normality of the data. Statistically significant differences between groups were determined using one‐way ANOVA. For multiple comparisons, Bonferroni's correction was applied for ≤4 groups and Tukey's correction was used for >4 groups, adjusting p‐values to reduce the probability of Type I errors. For behavioral tests described in the study, the number of mice analyzed in each group was specified in the respective figure legends. For other experiments, such as Western Blotting and Immunofluorescence, the definition of “n” was provided in the corresponding figure legends. All statistical details, including the exact value of “n”, what “n” represents, and the specific statistical test used, were also described in the figure legends. Additionally, relevant datasets were identified through database searches, including the RNA‐seq dataset for microglia (PRJNA613212), a diabetes‐related ChIP‐seq dataset (GSE51311), and a microglia‐specific ChIP‐seq dataset (GSE79812). These datasets enabled the analysis and visualization of correlations among genes involved in mannose, glucose, and fructose metabolism, as shown in Figure S1 (Supporting Information). The correlation coefficients among these metabolic genes across the three datasets were visualized using the corrplot package in R software, and the PerformanceAnalytics package was used to further refine and display these correlations.

## Conflict of Interest

The authors declare no conflict of interest.

## Author Contributions

L.L. and J.Z. conceptualized the study; C.L. and Z.Y. prepared and maintained mice; C.L., Z.Y., S.Y., Y.Z., Z.C., D.C., A.L., and H.L. designed and performed morphological analysis and biochemical assays. C.L., Z.Y., L.L., and Z.C. performed behavior tests. C.L., L.L. and J.Z. wrote the manuscript. C.L., L.L. and J.Z. discussed and edited the manuscript. J.Z. and L.L. supervised the project. All authors reviewed and gave final approval to the manuscript.

## Supporting information



Supporting Information

Supporting Information

## Data Availability

The data that support the findings of this study are available from the corresponding author upon reasonable request.
