# Supplementary material for: Mannose Promotes β‐Amyloid Pathology by Regulating BACE1 Glycosylation in Alzheimer's Disease
Source: Adv Sci (Weinh). 2025 Jan 14;12(9):2409105. doi: 10.1002/advs.202409105 (PMC11884605; doi:10.1002/advs.202409105)
Supplement: Supplementary file 1 — Supporting Information [file ADVS-12-2409105-s002.docx]

**Supplementary Information for**

**Mannose promotes β-amyloid pathology by regulating BACE1 glycosylation in Alzheimer’s disease**

*Chensi Liang^1,#^, Ziqi Yuan^1,#^, Shangchen Yang^1^, Yufei Zhu^1^, Zhenlei Chen^1^, Dan Can^1^, Aiyu Lei^1^, Huifang Li^1^, Lige Leng^1,2,3,^*, Jie Zhang^1,3,4,^**

**Inventory of Supplemental Information:**

Supplemental datasheet legend 1

Supplemental figures and figure legends:

Figure S1, related to Figure 1

Figure S2, related to Figure 2

Figure S3, related to Figure 2

Figure S4, related to Figure 3

Figure S5, related to Figure 4

Figure S6, related to Figure 5

Figure S7, related to Figure 5

**Dataset Legend:**

Datasheet 1. The differential metabolites of hippocampus of four group (6-month-old wildtype mice with standard diet and 5×FAD mice with standard diet; 5×FAD mice with standard diet and 5×FAD mice with mannose-free diet; 5×FAD mice with mannose-free diet and 5×FAD mice with mannose-free diet+10% mannose)

**Supplemental Figures and Figure Legends:**

**
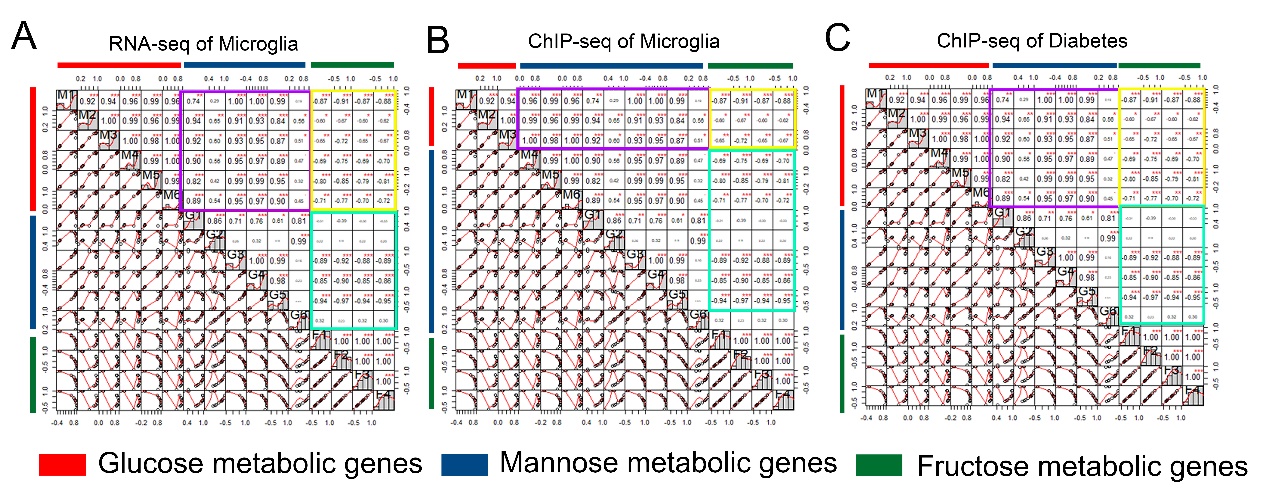
**

**Figure S1. Mannose levels are correlated with glucose levels. Related to Figure 1.**

(A-C) The corrplot package of R software is used to visualize the correlation coefficients of mannose, glucose and fructose metabolic genes in three databases, including RNA-seq database (PRJNA613212) (A), microglia chip-seq database (GSE79812) (B) and diabetes chip-seq database (GSE51311) (C). The Performance Analytics package of R software is used to visualize the correlation coefficients of mannose, glucose and fructose metabolic genes in three databases. The upper matrix shows the Pearson coefficients, and results were significant at *** p < 0.01, ** p < 0.05, or * p < 0.1 as marked. The red solid lines in the lower matrix show a smooth regression between the two factors. The diagonal is marked the distribution of the variable itself.

Data represent mean ± SEM, n.s.: not significant, *p<0.05, **p<0.01, ***p<0.001, one-way ANOVA with Tukey’s post hoc analysis.

**
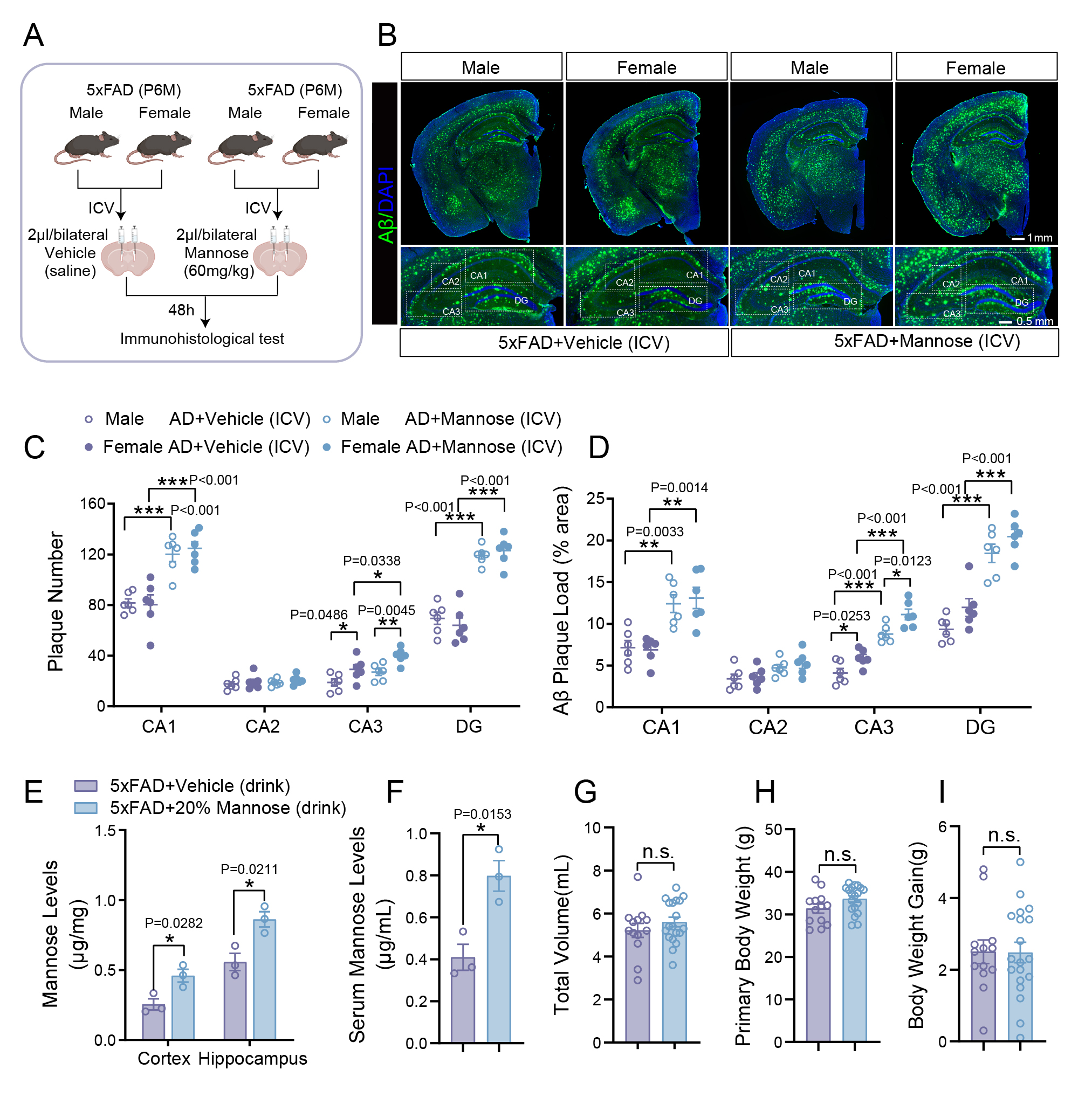
Figure S2. Sex-balanced study of mannose and physiological characters of 5×FAD mice drinking with different concentrations of mannose. Related to Figure 2.**

(A) Schematic diagram of ICV administration of mannose or vehicle in six male and six female 5×FAD mice, 48 hours after injection, the mice brain slice were subjected to immunostaining. (B-D) Immunofluorescence staining of Aβ in cerebrum from 6-month-old male and female 5×FAD mice treated with mannose or vehicle by ICV. Representative confocal images are shown on panel (B), Scale bar:1mm, 0.5mm. Quantitation of plaque number is showed in (C) and plaque load is showed in (D), n=6 mice. (E) The mannose levels in cortex and hippocampus of 5×FAD mice and 5×FAD drinking with 20% mannose were measured, n=3 mice. (F) The mannose levels in serum of 5×FAD mice and 5×FAD drinking with 20% mannose were measured, n=3 mice. (G) The water intake volume of 5×FAD mice and 5×FAD mice drinking with 20% mannose. (H) The primary body weight of 5×FAD mice and 5×FAD mice drinking with 20% mannose were measured. (I) The body weight gains of 5×FAD mice and 5×FAD mice drinking with 20% mannose.

There are two groups:

5×FAD mice: n=13;

5×FAD mice drinking 20% mannose: n=19.

Data represent mean ± SEM, n.s.: not significant, *p<0.05, **p<0.01, ***p<0.001, one-way ANOVA with Tukey’s post hoc analysis.

**
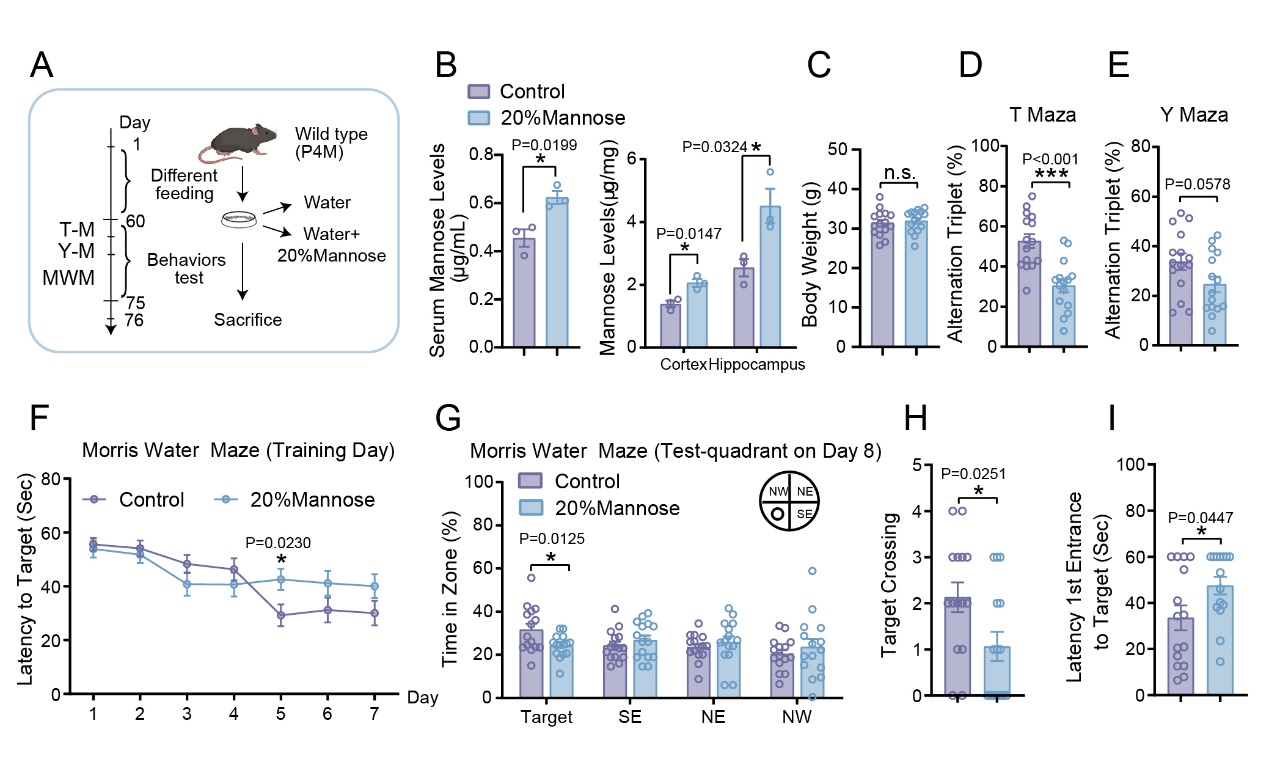
**

**Figure S3. Long-term intake of mannose could lead to cognitive decline in wild-type mice**

1. Schematic diagram of administration of 20% mannose in wild-type mice. (B) The mannose levels in serum, cortex and hippocampus of wild-type mice drinking water with or without 20% mannose were measured, n=3 mice. (C) The body weight of wild-type mice drinking water with or without 20% mannose were measured. (D) Behavioral analysis of 6-month-old wild-type mice drinking water with or without 20% mannose by T maze. (E) Behavioral analysis of 6-month-old wild-type mice drinking water with or without 20% mannose by Y maze. (F-I) Morris water maze tests of 6-month-old wild-type mice drinking water with or without 20% mannose were analyzed for escape latency during a 7-day training period (F), the time spent in the target zone and other quadrants (southwest, southeast, and northwest) (G), number of target crossings (H) and time required from entrance to the target platform (I) were measured on the next day.

Mouse number used in behavior tests:

wild-type mice: n=15 mice;

wild-type mice drinking 20% mannose: n=15 mice.

Data represent mean ± SEM, n.s.: not significant, *p<0.05, **p<0.01, ***p<0.001, unpaired t test for behavioral statistics. Other statistical applications were analyzed by one-way ANOVA with Tukey’s post hoc analysis.

**
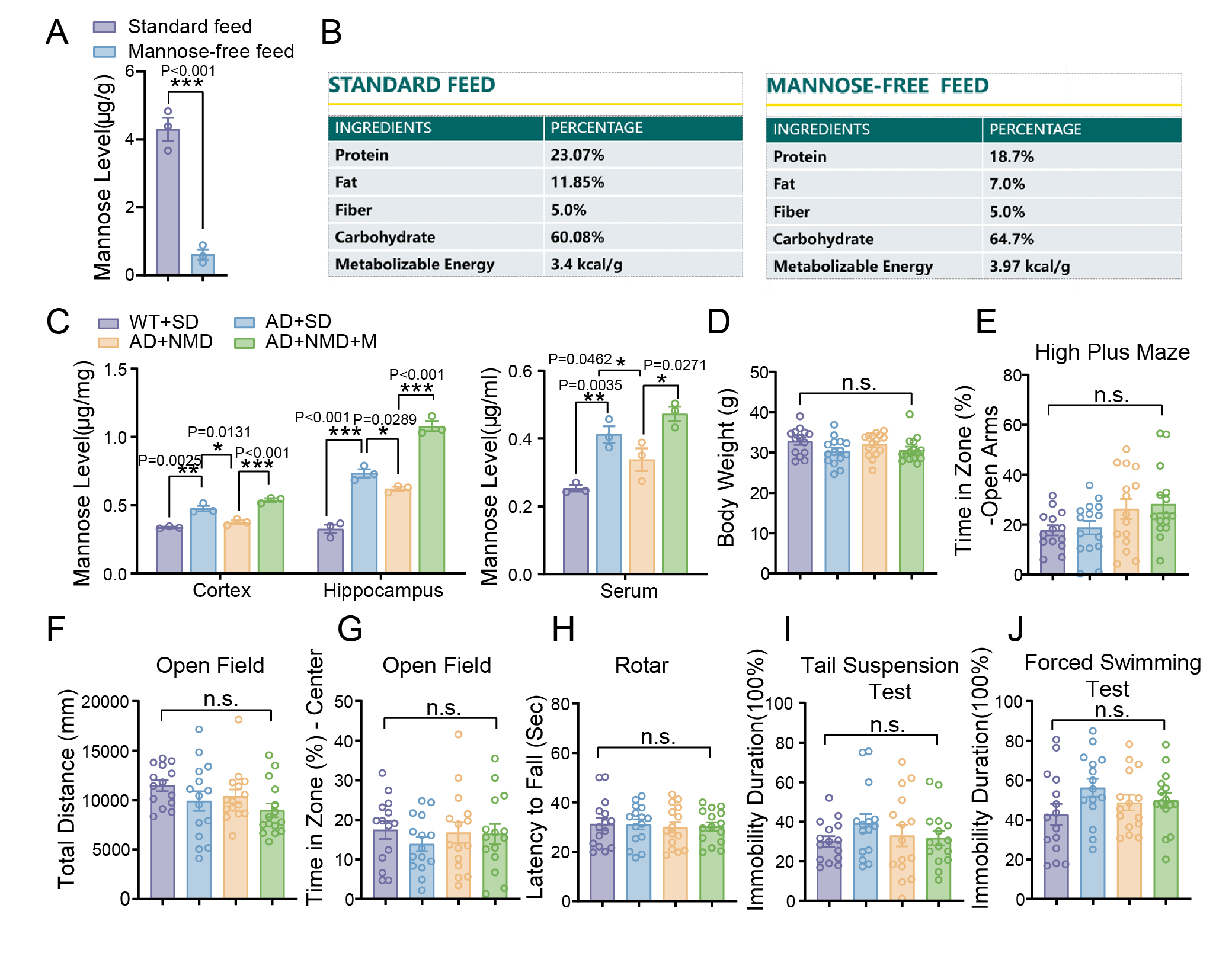
**

**Figure S4. Basic physiological characters and behavioral analysis of 5×FAD mice fed mannose-free and mannose-containing diets. Related to Figure 3.**

1. Mannose levels in standard food and mannose-free food were measured, n=3 experimental replication/group. (B) Ingredients of standard food and mannose-free food. (C) Mannose levels in cortex, hippocampus and serum from wildtype mice with standard diet (WT+SD), 5×FAD mice with standard diet (AD+SD), 5×FAD mice with no-mannose diet (AD+NMD) and 5×FAD mice with no-mannose diet+10% mannose (AD+NMD+M) were determined by ELISA, n=3 mice. (D) The body weight of above four group mice were measured. High plus maze (E), open field (F, G), rotarod test (H), tail suspension test (I) and forced swimming test (J) of 6-month-old WT+SD, AD+SD, AD+NMD and AD+NMD+M mice were measured.

There are four groups:

WT+SD: n=14 mice, AD+SD: n=14 mice, AD+NMD: n=15 mice, AD+NMD+M: n=15 mice.

Data represent mean ± SEM, n.s.: not significant, *p<0.05, **p<0.01, ***p<0.001, unpaired t test for behavioral statistics. Other statistical applications were analyzed by one-way ANOVA with Tukey’s post hoc analysis.

**
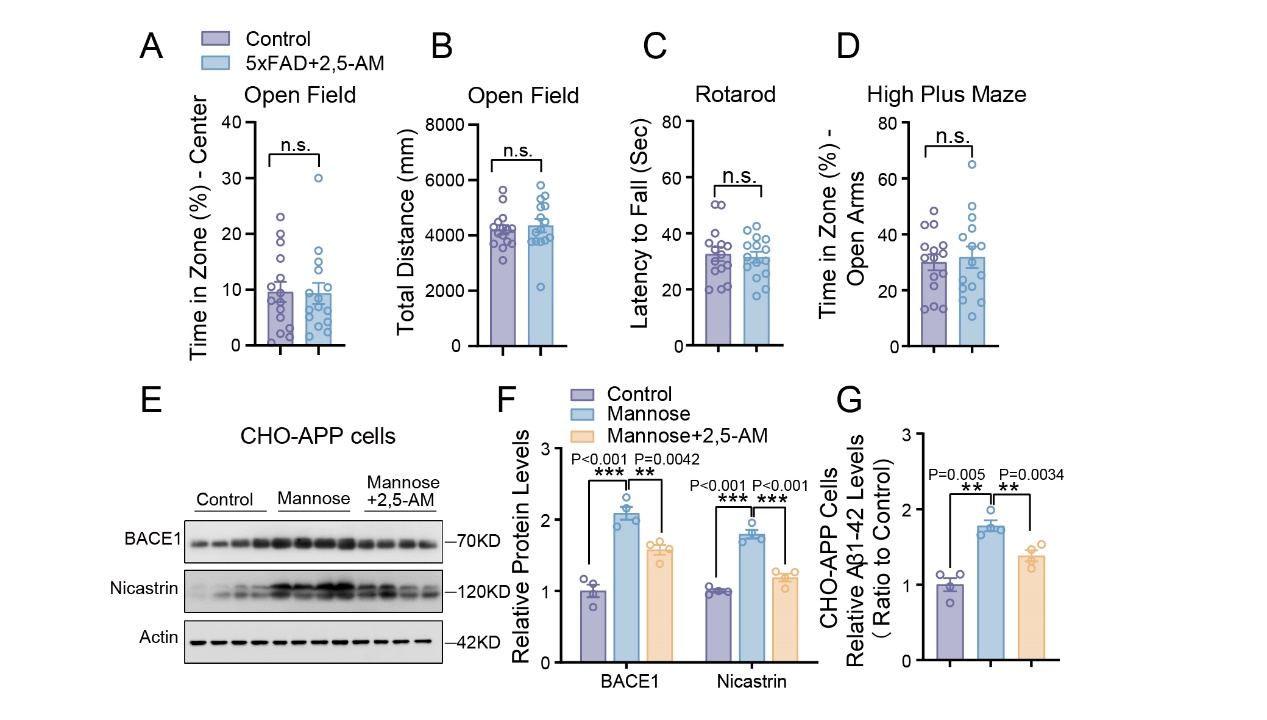
**

**Figure S5. Effects of 2,5-AM on motor function in AD mice and its pathological effects on CHO-APP cells *in vitro*. Related to Figure 4.**

1. D) Behavioral analysis of 6-month-old 5×FAD mice with or without 2,5-AM by open field (A, B), rotarod test (C) and high plus maze (D). (E, F) Western blot analysis of Bace1 and Nicastrin expression in CHO-APP cells treated with or without mannose and 2,5-AM. Quantification of relative proteins levels are showed in (F), n=4 experimental replication/group. (G) Aβ1-42 levels in different groups of CHO-APP cells were detected by ELISA kit. n=4 experimental replication/group.

Data represent mean ± SEM, n.s.: not significant, *p<0.05, **p<0.01, ***p<0.001, unpaired t test for behavioral statistics. Other statistical applications were analyzed by one-way ANOVA with Tukey’s post hoc analysis.


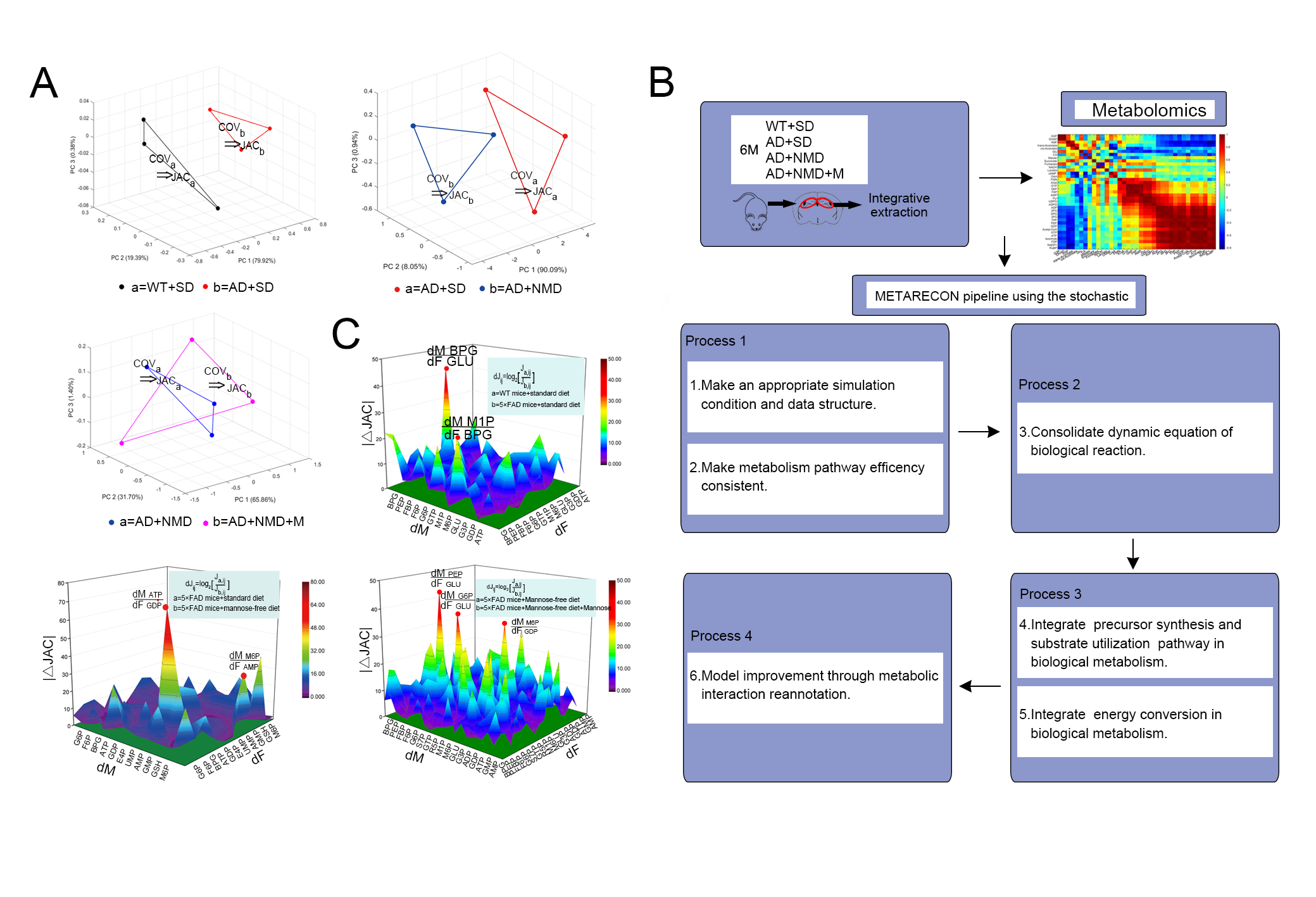


**Figure S6. Mannose affected the pathological process of 5×FAD through glycosylation modification. Related to Figure 5.**

(A) Differentially expressed metabolites were identified from hippocampus of 6-month-old WT+SD mice, AD+SD mice, AD+NMD mice and AD+NMD+M mice. Principal component analysis (PCA) of the metabolite data revealing a separation between the hippocampus of 6-month-old WT+SD and AD+SD mice; AD+SD and AD+NMD mice; AD+NMD and AD+NMD+M mice. The biological variance of the independent biological replicates per cell type is visible, which is further exploited first for the calculation of the Covariance matrix COV and subsequently for the Jacobian matrix JAC using the stochastic Lyapunov matrix Equation 1. (B) To allow for inverse modeling of biochemical regulation from metabolomics covariance data, a metabolic reconstruction and pathway reduction from available genome sequences is performed (RECON). (C) Differential Jacobian matrix of hippocampus of 6-month-old WT+SD mice, AD+SD mice, AD+NMD mice and AD+NMD+M mice BMDMs derived from covariance data from the metabolomics datasets. All entries represent median values of 10^3^ calculations normalized to the square of interquartile distance. df and dM characterize the entries of the Jacobian matrix and refer to Equation 2. The greatest perturbation between WT+SD and AD+SD mice was identified as the Jacobian entry *ðf_BPG_=ðf_GLU_*. The greatest perturbation between AD+SD and AD+NMD mice was identified as the Jacobian entry *ðf_ATP_=ðf_GDP_*, and the third perturbation was identified as the Jacobian entry *ðf_M6P_=ðf_AMP_*. The third perturbation between AD+NMD and AD+NMD+M mice was identified as the Jacobian entry *ðf_M6P_=ðf_GDP_*, pointing to glycosylation synthesis reactions in the underlying biochemical network.

**
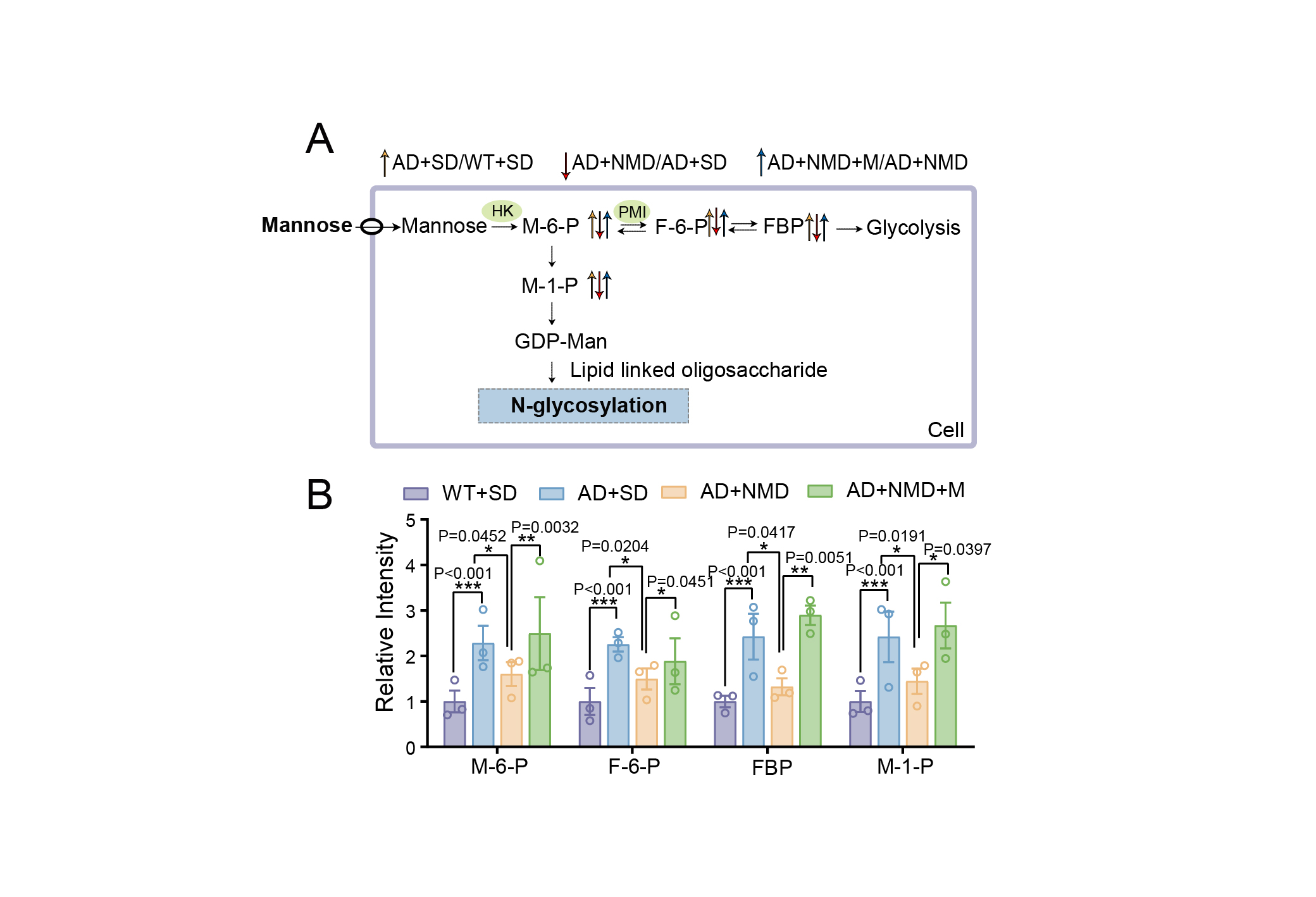
**

**Figure S7. Targeted metabolomics analysis of hippocampus of 6-month-old WT+SD, AD+SD, AD+NMD and AD+NMD+M mice. Related to Figure 5.**

(A) Schematic diagram of glycosylation pathway of mannose. (B) Metabolites of glycosylation pathway in hippocampus of 6-month-old WT+SD, AD+SD, AD+NMD and AD+NMD+M mice showed significant changes.

Data represent mean ± SEM, n.s.: not significant, *p<0.05, **p<0.01, ***p<0.001, unpaired t test for behavioral statistics. Other statistical applications were analyzed by one-way ANOVA with Tukey’s post hoc analysis.
